# Unprecedented N_2_O production by nitrate-ammonifying *Geobacteraceae* with distinctive N_2_O isotopocule signatures

**DOI:** 10.1128/mbio.02540-24

**Published:** 2024-10-30

**Authors:** Zhenxing Xu, Shohei Hattori, Yoko Masuda, Sakae Toyoda, Keisuke Koba, Pei Yu, Naohiro Yoshida, Zong-Jun Du, Keishi Senoo

**Affiliations:** 1 Marine College, Shandong University, Weihai, China; 2 Department of Applied Biological Chemistry, Graduate School of Agricultural and Life Sciences, The University of Tokyo, Tokyo, Japan; 3 International Center for Isotope Effects Research (ICIER), Nanjing University, Nanjing, China; 4 Frontiers Science Center for Critical Earth Material Cycling, State Key Laboratory for Mineral Deposits Research, School of Earth Sciences and Engineering, Nanjing University, Nanjing, China; 5 Collaborative Research Institute for Innovative Microbiology, The University of Tokyo, Tokyo, Japan; 6 School of Materials and Chemical Technology, Institute of Science Tokyo, Yokohama, Japan; 7 Center for Ecological Research, Kyoto University, Shiga, Japan; 8 SDU-ANU Joint Science College, Shandong University, Weihai, China; 9 Earth-Life Science Institute, Institute of Science Tokyo, Tokyo, Japan; 10 National Institute of Information and Communications Technology, Tokyo, Japan; University of Massachusetts Amherst, Amherst, Massachusetts, USA; University of Minnesota, St. Paul, Minnesota, USA

**Keywords:** nitrate-ammonifying bacteria, DNRA, *Geobacteraceae*, N_2_O production, N_2_O isotopocule signatures, paddy soils

## Abstract

Dissimilatory nitrate reduction to ammonium (DNRA), driven by nitrate-ammonifying bacteria, is an increasingly appreciated nitrogen-cycling pathway in terrestrial ecosystems. This process reportedly generates nitrous oxide (N_2_O), a strong greenhouse gas with ozone-depleting effects. However, it remains poorly understood how N_2_O is produced by environmental nitrate-ammonifiers and how to identify DNRA-derived N_2_O. In this study, we characterize two novel enzymatic pathways responsible for N_2_O production in *Geobacteraceae* strains, which are predominant nitrate-ammonifying bacteria in paddy soils. The first pathway involves a membrane-bound nitrate reductase (Nar) and a hybrid cluster protein complex (Hcp–Hcr) that catalyzes the conversion of NO_2_^−^ to NO and subsequently to N_2_O. The second pathway is observed in Nar-deficient bacteria, where the nitrite reductase (NrfA) generates NO, which is then reduced to N_2_O by Hcp–Hcr. These enzyme combinations are prevalent across the domain Bacteria. Moreover, we observe distinctive isotopocule signatures of DNRA-derived N_2_O from other established N_2_O production pathways, especially through the highest ^15^N-site preference (SP) values (43.0‰–49.9‰) reported so far, indicating a robust means for N_2_O source partitioning. Our findings demonstrate two novel N_2_O production pathways in DNRA that can be isotopically distinguished from other pathways.

**IMPORTANCE:**

Stimulation of DNRA is a promising strategy to improve fertilizer efficiency and reduce N_2_O emission in agriculture soils. This process converts water-leachable NO_3_^−^ and NO_2_^−^ into soil-adsorbable NH_4_^+^, thereby alleviating nitrogen loss and N_2_O emission resulting from denitrification. However, several studies have noted that DNRA can also be a source of N_2_O, contributing to global warming. This contribution is often masked by other N_2_O generation processes, leading to a limited understanding of DNRA as an N_2_O source. Our study reveals two widespread yet overlooked N_2_O production pathways in *Geobacteraceae*, the predominant DNRA bacteria in paddy soils, along with their distinctive isotopocule signatures. These findings offer novel insights into the role of the DNRA bacteria in N_2_O production and underscore the significance of N_2_O isotopocule signatures in microbial N_2_O source tracking.

## INTRODUCTION

**Fig 1:**
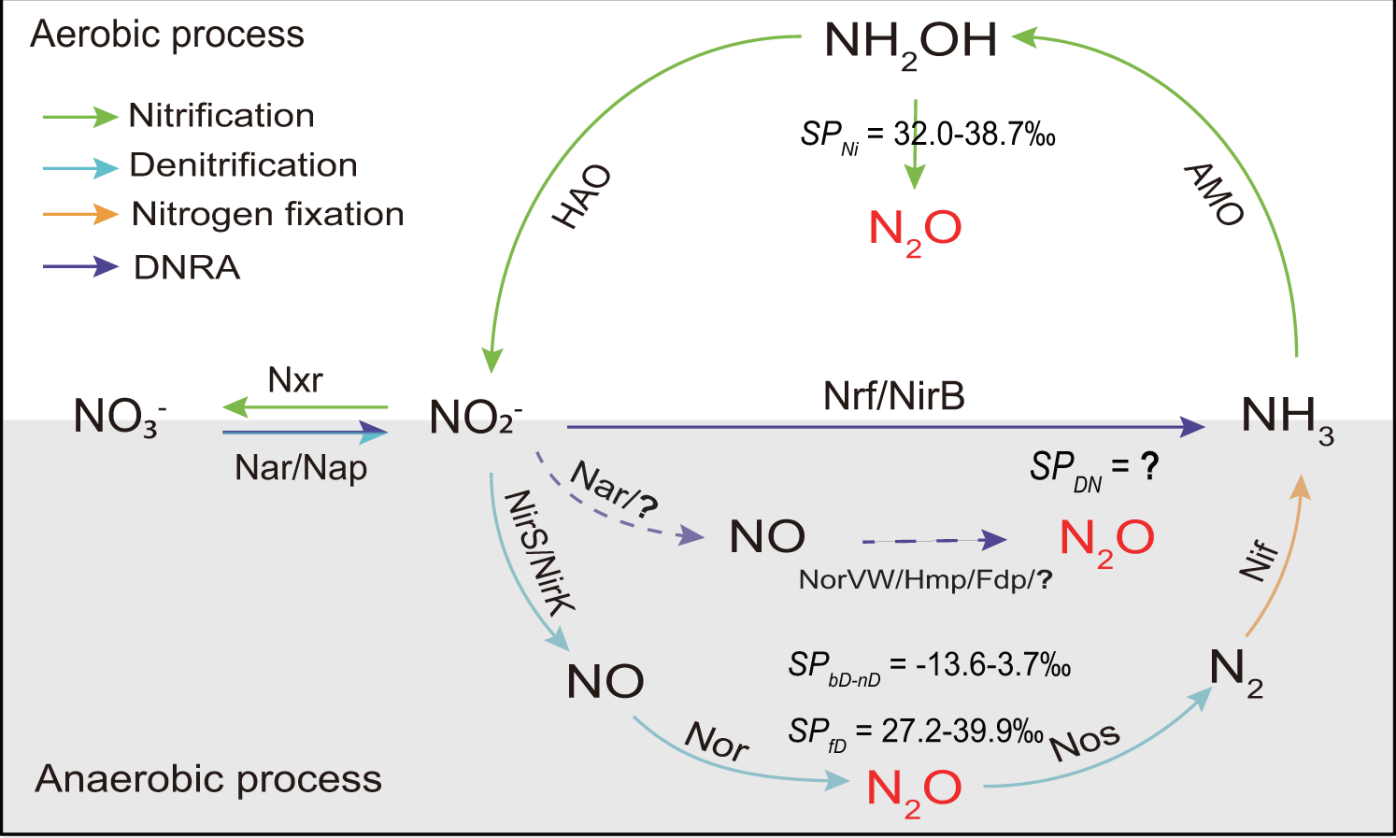
Simplified diagram showing N_2_O production pathways in the nitrogen cycle under both aerobic and anaerobic conditions. SP_bD_, SP_fD_, SP_NI_, and SP_DN_: ^15^N-site preference of N_2_O derived from the bacterial denitrification, fungal denitrification, nitrification, and DNRA processes, respectively. The value ranges of SP_bD_, SP_fD_, and SP_NI_ are retrieved from Ref. (9). Key enzymes of the nitrogen cycle are indicated as nitrate reductase (Nar/Nap), nitrite reductase (Nrf/NirB/NirS/NirK), NO dioxygenase (Hmp/Fdp), nitric oxide reductase (Nor), nitrous oxide reductase (Nos), nitrogenase (Nif), ammonia monooxygenase (AMO), hydroxylamine oxidoreductase (HAO), and nitrate oxidoreductase (Nxr). Dotted lines represent the pathways with predicted enzymes and intermediates.

Nitrous oxide (N_2_O) is a powerful greenhouse gas and the dominant ozone-depleting substance throughout the 21st century (1, 2). The largest current emissions of N_2_O into the atmosphere are driven by microbial activities in agricultural soils, exacerbated by the application of nitrogen fertilizers (3). Denitrification and nitrification are well-established as the dominant processes, accounting for approximately two-thirds of all soil-derived N_2_O emissions (4). However, another microbial process, dissimilatory nitrate reduction to ammonium (DNRA), has also been implicated in contributing to global N_2_O emission (5, 6). DNRA, also termed nitrate (NO_3_^−^) ammonification, is a nitrogen (N)-retaining process in which water-leachable NO_3_^-^ is anaerobically reduced to soil-adsorbable NH_4_^+^ *via* the intermediate nitrite (NO_2_^−^). This reduction is catalyzed by cytoplasmic and/or periplasmic NO_3_^−^ reductases (Nar and/or Nap) and cytochrome *c_552_* and/or NADH-dependent NO_2_^−^ reductases (NrfA and/or NirB) in a two-step process (Fig. 1)(7, 8).

The phenotype of N_2_O production in DNRA was first documented in 1981, where 163 out of 168 studied nitrate-ammonifying soil bacteria were found to produce N_2_O (10). Subsequent research has identified many nitrate-ammonifiers, primarily affiliated with *Proteobacteria* and the genus *Bacillus*, as N_2_O producers (11–13). The widely accepted pathway for N_2_O production in these bacteria involves the generation of N₂O from NO₂^−^ *via* a two-step process, with NO as an intermediate. This process is catalyzed by Nar and NO detoxification-related enzymes, including flavodiiron proteins (Fdp), flavorubredoxin (NorVW), and flavohemoglobin (Hmp), as demonstrated in Nar-containing bacteria *Escherichia coli* and *Salmonella typhimurium* (Fig. 1) (14–18). However, several studies have reported contradictory findings. For instance, the Hmp and NorV mutations in *E. coli* did not impair NO reduction (19). Additionally, a recent study involving soil DNRA bacteria suggested that N_2_O production from Nap and Nar was unlikely, with the NO_2_^−^-to-NH_4_^+^ reaction being a more plausible N_2_O source (20). This raises questions about the mechanism of N_2_O generation in Nar-containing ammonifiers. To date, there has been no attempt to explore the N_2_O generation mechanism in Nap-driven nitrate-ammonifiers (Nar-absent), although their phenotype of N_2_O production from NO_3_^−^ has been observed in the DNRA model organism *Wolinella succinogenes* (21). Therefore, a comprehensive understanding of DNRA-derived N_2_O production requires further investigation of DNRA bacteria that use Nar versus Nap enzymes for NO_3_^−^ reduction.

The analysis of N_2_O isotopocule signatures (δ^15^N^bulk^, δ^18^O, and ^15^N-site preference [SP]) is a promising tool for tracing the N_2_O origins and quantifying the contributions of distinct production processes, as they rely on the natural abundance of N and O isotopes without perturbing *in situ* conditions (22). Particularly, SP refers to the difference in the ^15^N isotopic composition of ^α^N compared with the ^β^N in the linear N_2_O molecule (^β^N-^α^N-O)(22). Unlike conventional isotopic modes like δ^15^N^bulk^ and δ^18^O, SP is independent of the concentrations and isotope ratios of substrates, making it a unique indicator for distinguishing N_2_O production pathways (9). Although the N₂O isotopocule signatures of most known N₂O generation pathways have been documented (Fig. 1) (9, 23), those associated with DNRA remain unexplored. N_2_O production *via* DNRA and other pathways, especially denitrification, often occurs simultaneously under anaerobic conditions (6, 24), but few studies have evaluated the contribution of DNRA to N_2_O emissions due to the lack of a means for distinguishing DNRA-derived N_2_O from bulk N_2_O emissions. Characterizing the N_2_O isotopocule signatures associated with DNRA could enable the partitioning of bulk N₂O emissions into contributions from DNRA and other pathways.

**Fig 2:**
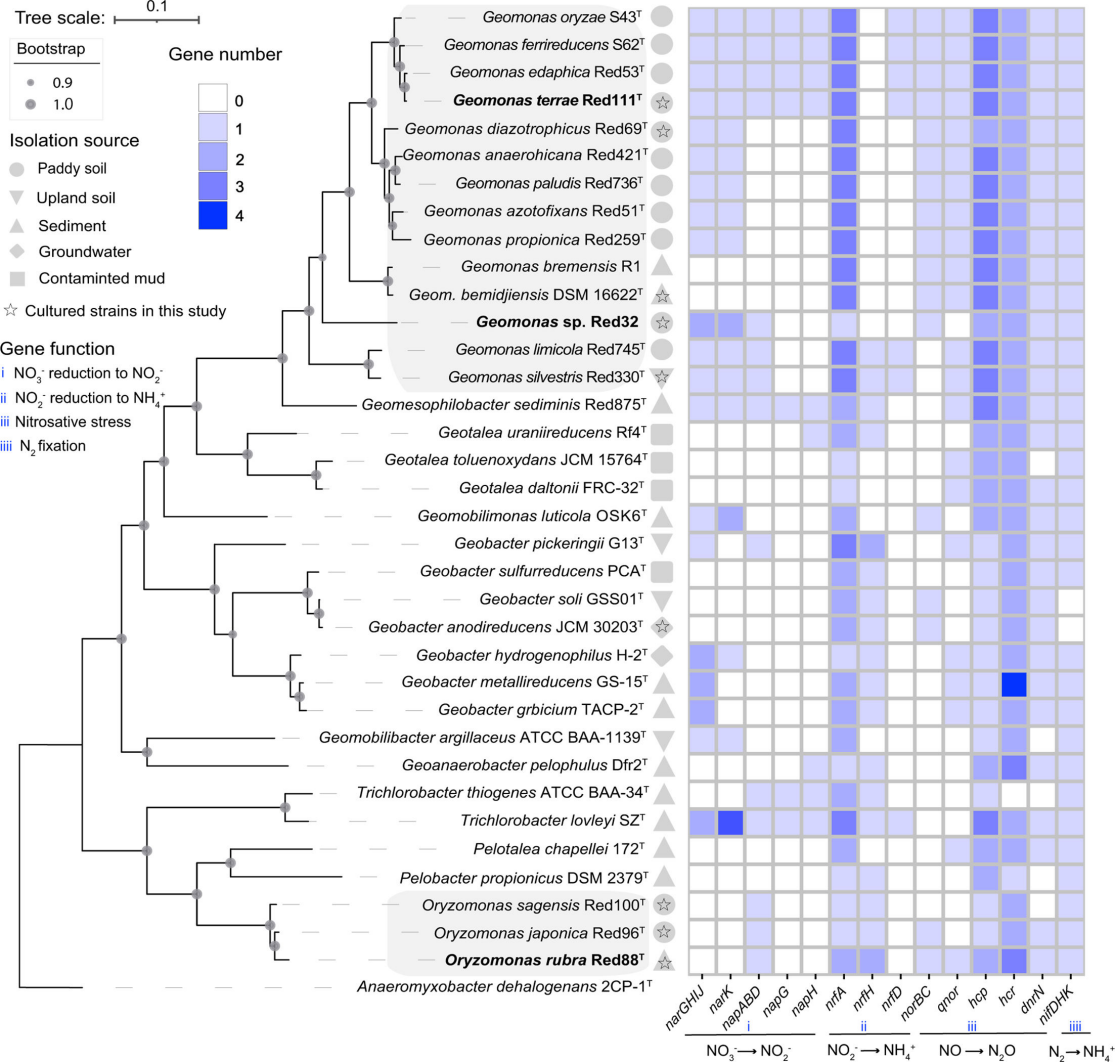
Phylogenomics and nitrogen metabolism-related gene inventory of *Geobacteraceae* species. The tree is a maximum-likelihood (ML) phylogeny based on 92 concatenated core proteins in the UBCG database (35). Bootstrap values shown in circle shapes at branching nodes were calculated using 100 replicates. The scale bar represents 0.1 substitutions per amino acid position. The studied genera *Geomonas* and *Oryzomonas* were labelled by gray color. The nitrogen metabolism-related gene inventory is shown in the heatmap and the copy numbers of every functional gene are denoted by the blue bar. The accession numbers of genome sequences used in tree construction and detailed information of the strain habitat are listed in Table S4.

Paddy soils are the largest anthropogenic wetlands on Earth and contribute greatly to global N_2_O emissions (25, 26). These soils are rich in DNRA drivers due to the reducing conditions caused by waterlogging during rice cultivation (27). *Geobacteraceae* is a strictly anaerobic and ubiquitous bacterial group in terrestrial ecosystems (28) and one of the dominant nitrate-ammonifying groups in paddy soils (27, 29). Recently, we isolated dozens of *Geomonas* and *Oryzomonas* strains (both belonging to the family *Geobacteraceae*) from paddy soils and nearby sediments (30–34). They were genome-annotated as different types of nitrate-ammonifiers, as *Geomonas* strains mainly contain only Nar or both Nar and Nap for NO_3_^−^ reduction, while *Oryzomonas* strains only possess Nap (Fig. 2). The co-presence of Nar- and Nap- driven strains in these closely related genera provides an ideal model for investigating the diverse features of DNRA, including the N_2_O generation mechanisms.

In this study, we investigated the N_2_O production pathways and the key enzymes involved in strictly anaerobic nitrate-ammonifiers *Geobacteraceae* and synchronously measured the isotopocule signatures of DNRA-derived N_2_O. We hypothesized that these nitrate-ammonifiers would exhibit novel N_2_O production pathways or N_2_O-generating enzymes, given the absence of *fdp*, *norVW*, and *hmp* genes related to NO detoxification reported in enteric facultative anaerobes (16–18), and thus possibly create distinctive N_2_O isotopocule signatures. This study provides a comprehensive description of DNRA-derived N_2_O production in environmental microorganisms, enhancing our understanding of N_2_O emission pathways and the role of DNRA microorganisms in greenhouse gas production.

## RESULTS AND DISCUSSION

### DNRA activity and N_2_O production in representative *Geomonas* and *Oryzomonas* strains

*Geomonas* and *Oryzomonas* species were genome-annotated as nitrate-ammonifiers, as they contain the key marker genes *nar*/*nap* and *nrfA* of the DNRA, except for two strains, *Geomonas bremensis* R1 and *Geomonas bemidjiensis* DSM 16622^T^, which lack *nar* and *nap* genes and are solely nitrite-ammonifiers (Fig. 2). These strains exhibit various combinations of functional genes related to N metabolism, but these combinations are conserved within phylogenetic clusters at the species level (Fig. 2). We selected three representative species, including two Nar-containing bacteria *Geomonas* sp. Red32 and *Geomonas terrae* Red111 and one Nar-absent but Nap-containing bacterium *Oryzomonas rubra* Red88 to assay DNRA activities using either NO_3_^−^ (8 mM) or NO_2_^−^ (2 mM) as substrates.

**Fig 3:**
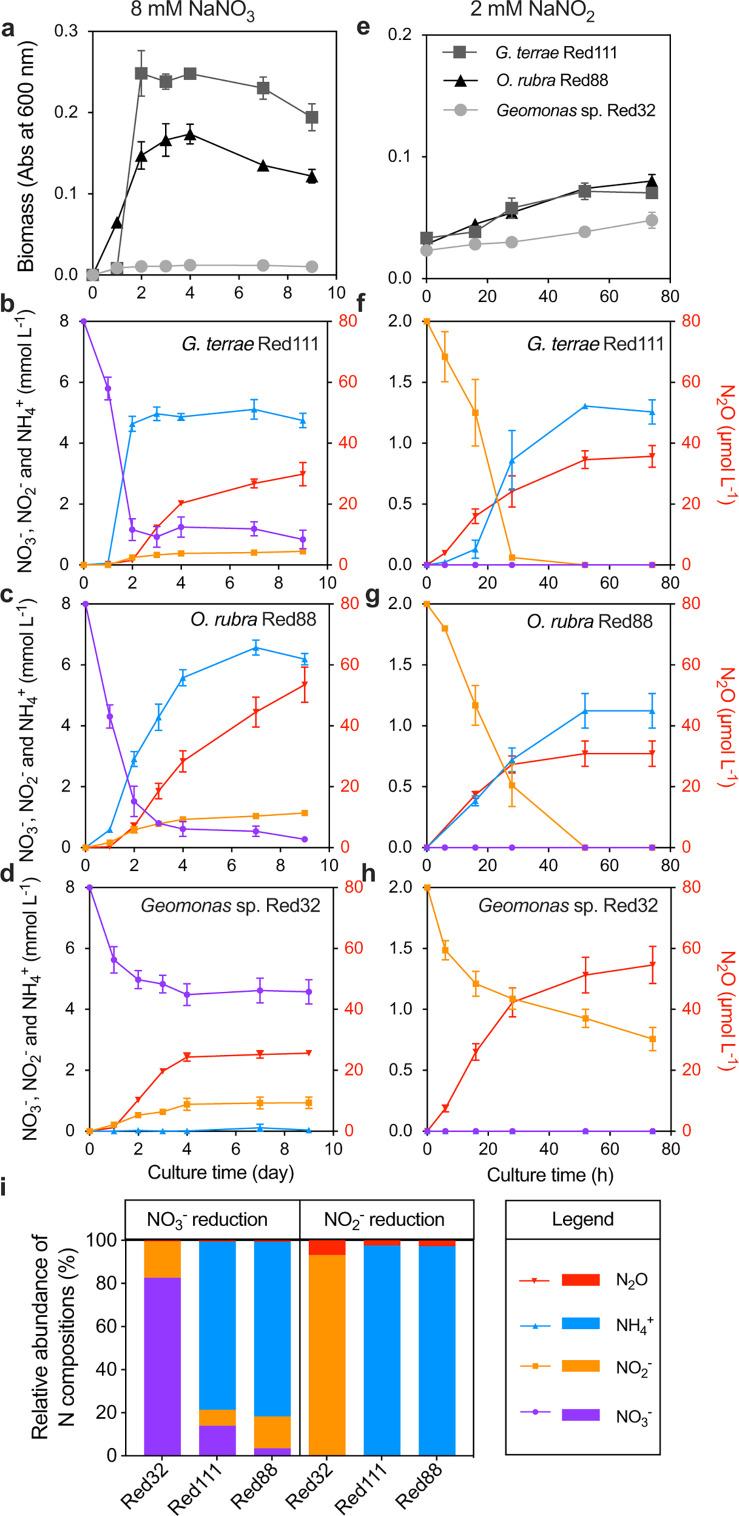
Dissimilatory NO_3_^−^ and NO_2_^−^ reduction by three *Geobacteraceae* strains. (**a–h**) Dynamics of growth conditions and concentrations of NO_3_^−^, NO_2_^−^, NH_4_^+^, and N_2_O in the cultures after NO_3_^−^ (8 mM, left panel) or NO_2_^−^ (2 mM, right panel) additions for *Geomonas terrae* Red111, *Oryzomonas rubra* Red88, and *Geomonas* sp. Red32. The start time was from the bacterial inoculation for the NO_3_^−^ reduction process and from the NO_2_^−^ addition for the NO_2_^−^ reduction process. The values are shown as mean ± standard deviation (SD). (**i**) The relative abundance of N compositions in the cultures at the final reaction stage during NO_3_^−^ and NO_2_^−^ reduction by the three strains.

*G. terrae* Red111 and *O. rubra* Red88 were observed to utilize NO_3_^−^ for growth, producing NH_4_^+^, NO_2_^−^, and N_2_O as end products, thereby demonstrating DNRA activity (Fig. 3a through c). In contrast, *Geomonas* sp. Red32 produced only NO_2_^−^ and N_2_O without NH_4_^+^ production or significant biomass increase (Fig. 3a and d), likely due to its weak NO_2_^−^-reducing activity, which may be attributed to the single copy of the *nrfA* gene (Fig. 2). When NO_2_^−^ was the substrate, *G. terrae* Red111 and *O. rubra* Red88 completely consumed NO_2_^−^, producing NH_4_^+^ and N_2_O along with bacterial growth (Fig. 3e through g). In contrast, *Geomonas* sp. Red32 only partially consumed NO_2_^−^, producing N_2_O but no NH_4_^+^ (Fig. 3e and h). None of the studied strains produced NO_3_^−^ from the added NO_2_^−^ (Fig. 3f through h), indicating that NO_2_^−^ oxidation did not occur in any of the cultures. Notably, the N_2_O produced by these nitrate-ammonifiers accounted for only 1% or less of consumed NO_3_^−^ and 1%–3% of consumed NO_2_^−^, far less than the main product NH_4_^+^ (>60% of substrates for *G. terrae* Red111 and *O. rubra* Red88) and the accumulated NO_2_^−^ during NO_3_^−^ reduction (3%–20%) (Fig. 3i). This suggests that N_2_O production during DNRA is a byproduct rather than a key intermediate or main product.

The facultative anaerobic nitrate-ammonifiers have been reported to produce N_2_O at levels accounting for 3%–36% of consumed NO_3_^−^ (10, 36, 37), indicating that these *Geobacteraceae* strains have a relatively low N_2_O production ratio from NO_3_^−^ transformation. However, such ratios are comparable to those observed in the strict anaerobe *Wolinella succinogenes*, which converted approximately 0.15% of NO_3_^−^ to N_2_O *via* DNRA (21). This finding indicates a difference in N_2_O production capacities between strictly and facultative anaerobic nitrate-ammonifiers, possibly resulting from the difference in the pathways or enzymes involved in N_2_O production. Moreover, among the biological processes of N_2_O production, denitrification exhibits the highest N_2_O production ratio from NO_3_^−^, as N_2_O is a key intermediate and can be the sole product, accounting for all consumed NO_3_^−^, depending on culture conditions and denitrifier types (38). Previous studies have reported that ammonia-oxidizing bacteria (AOB) converted 0.3%–10% of substrate NH_3_ to N_2_O and its precursor NO during both NH_3_ oxidation to NO_2_^−^ and NO_2_^−^ reduction to N_2_O (39). In contrast, ammonia-oxidizing archaea (AOA) and comammox bacteria yield approximately 0.08% N_2_O from substrate NH_3_ (40, 41), and anammox bacteria converted 0.1% of substrates NO_2_^−^ and NH_4_^+^ to N_2_O (42). These findings suggest that nitrate-ammonifiers produce N_2_O at levels comparable to AOB, and much higher than AOA and comammox and anammox bacteria. Given the wide distribution and diverse species of nitrate-ammonifying bacteria (6), DNRA has great potentials as a contributor to greenhouse gas emission and should not be overlooked.

### N_2_O production coupled to NO_2_^−^ and NO accumulation during DNRA

**Fig 4:**
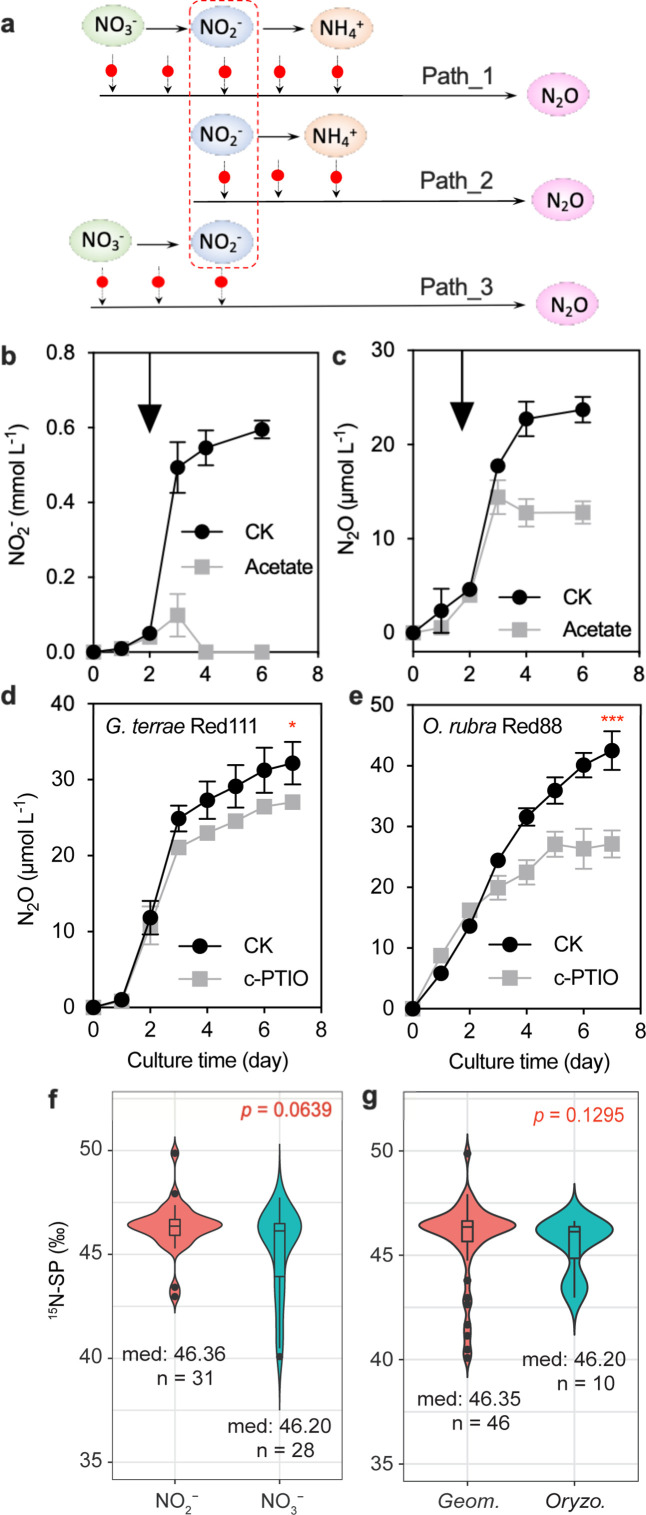
Comparison of N_2_O production and SP values showing the unique N_2_O production from NO_2_^−^ *via* the intermediate NO. (**a**) A comparison diagram showing the possible N_2_O production source on DNRA. Three pathways, labeled by Path_1, Path_2, and Path_3, represent the experimental results from the NO_3_^−^ reduction by all studied *Geomonas* strains except for Red32, NO_2_^−^ reduction by all studied *Geomonas* strains except for Red32, and NO_3_^−^ reduction by strain Red32, respectively. The red dots on the arrows indicate the possible source of N_2_O production. (**b and c**) Dynamics of NO_2_^−^ and N_2_O concentrations during the NO_3_^−^ reduction process driving by *Geomonas terrae* Red111 with (gray squares) and without (black circles) acetate (5 mM) addition. Arrows indicate the time when the extra acetate was added to the medium. (**d and e**) N_2_O production from the NO_3_^−^ reduction process driven by *Geomonas* strain Red111 and *Oryzomonas* strain Red88 across time with (gray squares) and without (black circles) c-PTIO additions. c-PTIO was added to the medium at the same time to bacteria inoculation. CK indicates control experiments, asterisks indicate significant difference, *** *P* < 0.001, *P < 0.05, ns indicates insignificant difference, *P* > 0.05. The values are shown as mean ± standard deviation (SD). (**f and g**) The comparison of SP values between different genera (*Geomonas* and *Oryzomonas*) and substrates (NO_3_^−^ and NO_2_^−^). Med and n indicate the median and number of every data set, respectively.

To determine the direct substrate contributing to N_2_O during DNRA, we conducted a comparative analysis of N_2_O production across different strains and substrates based on prior culture experiments. First, N_2_O production from NO_3_^−^ in *G. terrae* Red111 and *O. rubra* Red88 suggested five potential sources for N_2_O: NO_3_^−^, NO_2_^−^, NH_4_^+^, and the intermediate reactions between NO_3_^−^ and NO_2_^−^, and between NO_2_^−^ and NH_4_^+^ (Fig. 3b and c; Path_1 in Fig. 4a). Given that N_2_O production also occurred from NO_2_^−^ in *G. terrae* Red111 and *O. rubra* Red88 (Fig. 3f and g), we ruled out NO_3_^−^ and the intermediate processes between NO_3_^−^ and NO_2_^−^ as N_2_O sources (Path_2 in Fig. 4a). Then, *Geomonas* sp. Red32 partially consumed NO_3_^−^ and NO_2_^−^ with concurrent N_2_O generation, but no NH_4_^+^ was detected in the medium (Fig. 3d and h), eliminating NH_4_^+^ and the intermediate process between NO_2_^−^ and NH_4_^+^ as N_2_O sources (Path_3 in Fig. 4a). Collectively, NO_2_^−^ remains the most likely substrate for N_2_O (or its precursor) production during DNRA (Fig. 4a).

To verify the relationship between NO_2_^−^ and N_2_O in DNRA, we compared the production and consumption rates of N_2_O and other N compounds in cultures. N_2_O production was slower than NO_3_^−^ consumption and NH_4_^+^ production (Fig. 3b and c) but comparable to NO_2_^−^ accumulation with significantly positive linear relations (R^2^>0.7, *P* < 0.0001) (Fig. S1). Additionally, more carbon sources (high C:N ratios) were found to reduce the NO_2_^−^ accumulation during NO_3_^−^ reduction in *G. terrae* Red111 (Fig. S2). In a further study, the absence of NO_2_^−^ accumulation halted N_2_O production (Fig. 4b and c). These results indicate that NO_2_^−^ is the direct substrate contributing to N_2_O production (or its precursor) during DNRA in *Geobacteraceae* strains, consistent with previous findings that N_2_O is generated from NO_2_^−^ in nitrate-ammonifying *Enterobacteriaceae* (15, 16, 36).

Previous studies have reported that NO is produced before N_2_O in nitrate-ammonifying *Enterobacteriaceae* (14–18), raising the question of whether intermediates, such as NO and hydroxylamine, exist between NO_2_^−^ and N_2_O in *Geobacteraceae* strains. We measured hydroxylamine concentrations in the cultures and used NO inhibitors (c-PTIO and L-NMMA) and donors (SNP) for treatments. c-PTIO, a NO scavenger, removes NO from the medium, while L-NMMA, a NO synthase inhibitor, prevents endogenous NO generation from amino acids (43, 44). Hydroxylamine was undetected during bacterial growth, but c-PTIO (100 µM) significantly inhibited N_2_O production (*P* < 0.05; Fig. 4d and e). Notably, c-PTIO had no impact on bacterial growth or the DNRA rate, even at high concentrations of 300 µM (*P* > 0.05, Fig. S3), and L-NMMA (100 µM) did not affect N_2_O production (*P* > 0.05, Fig. S4). These results indicate that c-PTIO inhibits N_2_O production by reducing exogenous NO (derived from supplied NO_3_^−^) rather than by affecting bacterial activity. Furthermore, *G. terrae* Red111 and *O. rubra* Red88 converted approximately half of the supplied extra NO (80 µM SNP solution) to N_2_O (Fig. S5), demonstrating their ability to reduce NO to N_2_O. Altogether, we believe that NO is an intermediate between NO_2_^−^ and N_2_O, contributing partially or entirely to N_2_O production during DNRA. Notably, c-PTIO reduced N_2_O emission by 39.9% in *Oryzomonas* strains, but only by 19.8% in *Geomonas* strains (Fig. 4b and c). This difference suggests that *Oryzomonas* and *Geomonas* strains probably contain different pathways or enzymes for converting NO_3_^−^ to N_2_O.

The process proposed for N_2_O production from NO_2_^−^ *via* NO during DNRA is plausible for *Geobacteraceae* strains. However, three questions remain unanswered: 1) Is NO_2_^−^ the sole substrate contributing to N_2_O in all studied strains? 2) Are there other N_2_O production pathways besides the NO-mediated one? 3) Does N_2_O reduction occur during N_2_O generation in the studied strains? To address these questions, we measured and analyzed SP values, which depend solely on the structures of enzymatic precursors right before N_2_O production (22). The SP values of N_2_O produced from NO_3_^−^ ranged from 44.8‰ to 47.7‰ in 3-day cultures and from 40.1‰ to 46.6‰ in 7-day cultures, while those from NO_2_^−^ ranged from 43.4‰ to 49.9‰ in 3-day cultures and from 43.0‰ to 47.3‰ in 7-day cultures (Table 1; Table S1). Comparing different substrates, the median SP values for NO_2_^−^- and NO_3_^−^- derived N_2_O were 46.2‰ and 46.4‰, respectively, with no significant difference (*P* > 0.05) (Fig. 4f). Similarly, the SP values were insignificant (*P* > 0.05) among strains in the two genera *Geomonas* and *Oryzomonas*, with close medians of 46.4‰ and 46.2‰, respectively (Fig. 4g). These similar SP values indicate a shared N_2_O production pathway in these strains, regardless of whether NO_2_^−^ or NO_3_^−^ is used as the substrate. Additionally, to prove the uniqueness of the N_2_O production pathway, we compared the SP values of N_2_O collected at different reaction times, 3 and 7 days. *Geomonas* sp. Red32 and *O. rubra* Red88 showed constant SP values for both NO_3_^−^ and NO_2_^−^ reduction processes, whereas *G. terrae* Red111 only showed consistent SP values for NO_2_^−^ reduction (Table 1). It is known that mixed N_2_O production/reduction pathways can alter their relative contribution ratios over time, thereby changing SP values. However, the consistent SP values observed in this study refute this proposal and demonstrate a single, consistent pathway for N_2_O generation in the studied strains, except for NO_3_^−^ reduction driven by *G. terrae* Red111, where mixed processes for N_2_O production may occur (see the last section for explanation). These findings prove a unique N_2_O production pathway (NO_2_^−^-NO-N_2_O) without N_2_O reduction in the studied nitrate-ammonifiers, addressing the questions above.

**TABLE 1:** Isotopocule signatures of N_2_O produced from NO_3_^−^ and NO_2_^−^ reduction processes by *Geobacteraceae* strains and abiotic reactions^*a*^

Substrate	Strain^*b*^	Incubation time (d)^*c*^	δ^18^O (‰)	δ^15^N^bulk^ (‰)	SP (‰)
NO_3_^-^	*Geomonas* sp. Red32	3	16.2 (0.1)	−39.8 (0)	46.5 (0.1)
(8 mM)		7	14.6 (0.4)	−27.5 (1.2)	45.4 (1.4)
	*G. diazotrophica* Red69	3	26.4 (4.1)	−29.6 (4.5)	45.5 (0.7)
		7	23.6 (3.9)	−28.2 (4.9)	41.9 (0.9)
	*G. terrae* Red111	3	15.2 (1.2)	−19.7 (2.1)	47.0 (0.3)
		7	9.6 (0.7)	−23.0 (3.4)	41.1 (1.4)
	*G. silvestris* Red330	3	18.6 (4.1)	−21.1 (7.0)	46.8 (1.3)
		7	15.5 (1.5)	−18.0 (7.2)	46.5 (0.1)
	*O. rubra* Red88	3	23.4 (0.8)	−31.4 (3.7)	46.3 (0.6)
		7	19.2 (1.5)	−22.4 (5.4)	46.4 (0.3)
NO_2_^-^	*Geomonas* sp. Red32	3	27.8 (0.2)	−23.7 (0)	46.8 (0.2)
(2 mM)		7	27.0 (1.0)	−21.3 (0.1)	46.1 (1.0)
	*G. diazotrophica* Red69	3	28.8 (0.1)	−23.7 (0)	45.8 (0.7)
		7	28.6 (0)	−23.5 (0.3)	46.0 (0.1)
	*G. terrae* Red111	3	27.5 (0.3)	−24.3 (0.1)	47.2 (0.7)
		7	29.5 (0)	−23.9 (0.1)	46.3 (0.2)
	*G. silvestris* Red330	3	28.5 (0.4)	−22.5 (0.5)	46.5 (0.2)
		7	28.6 (0.2)	−21.6 (0.5)	46.4 (0.2)
	*O. rubra* Red88	3	28.8 (0.4)	−21.6 (0.7)	44.8 (2.0)
		7	28.8 (0.3)	−21.0 (0.1)	44.4 (2.0)
	*G. bemidjiensis* DSM 16622	3	28.6 (0.6)	−22.9 (0.2)	47.4 (2.3)
	*Geob. anodireducens* JCM 30203	3	28.7 (0.7)	−23.0 (0)	46.7 (0.7)
	Abiotic	1	20.1 (0.2)	−28.8 (0.4)	20.0 (1.2)

^*a*^ All N_2_O isotope values are the isotopic composition of the N_2_O produced, the δ^15^N and δ^18^O values are the difference from those of the substrate NO_3_^−^ (δ^15^N_NO3-_ = 2.4 ± 0.1‰, δ^18^O_NO3-_ = 17.9 ± 0.3‰) or NO_2_^−^ (δ^15^N_NO2-_ = −1.8 ± 0.3‰, δ^18^O_NO2-_ = 3.7 ± 0.2‰). The values in parentheses represent the standard deviation obtained from replicate experiments (refer to the raw data in Table S1).

^*b*^ Abiotic represents the N_2_O production from a chemical reaction without bacteria inoculation under low pH conditions (pH < 2).

^*c*^ Sample collection at different incubation times, the start time is from that the substrate NO_3_^−^ or NO_2_^−^ was injected into the cultures.

### Enzymatic pathways of N_2_O production during DNRA

**Fig 5:**
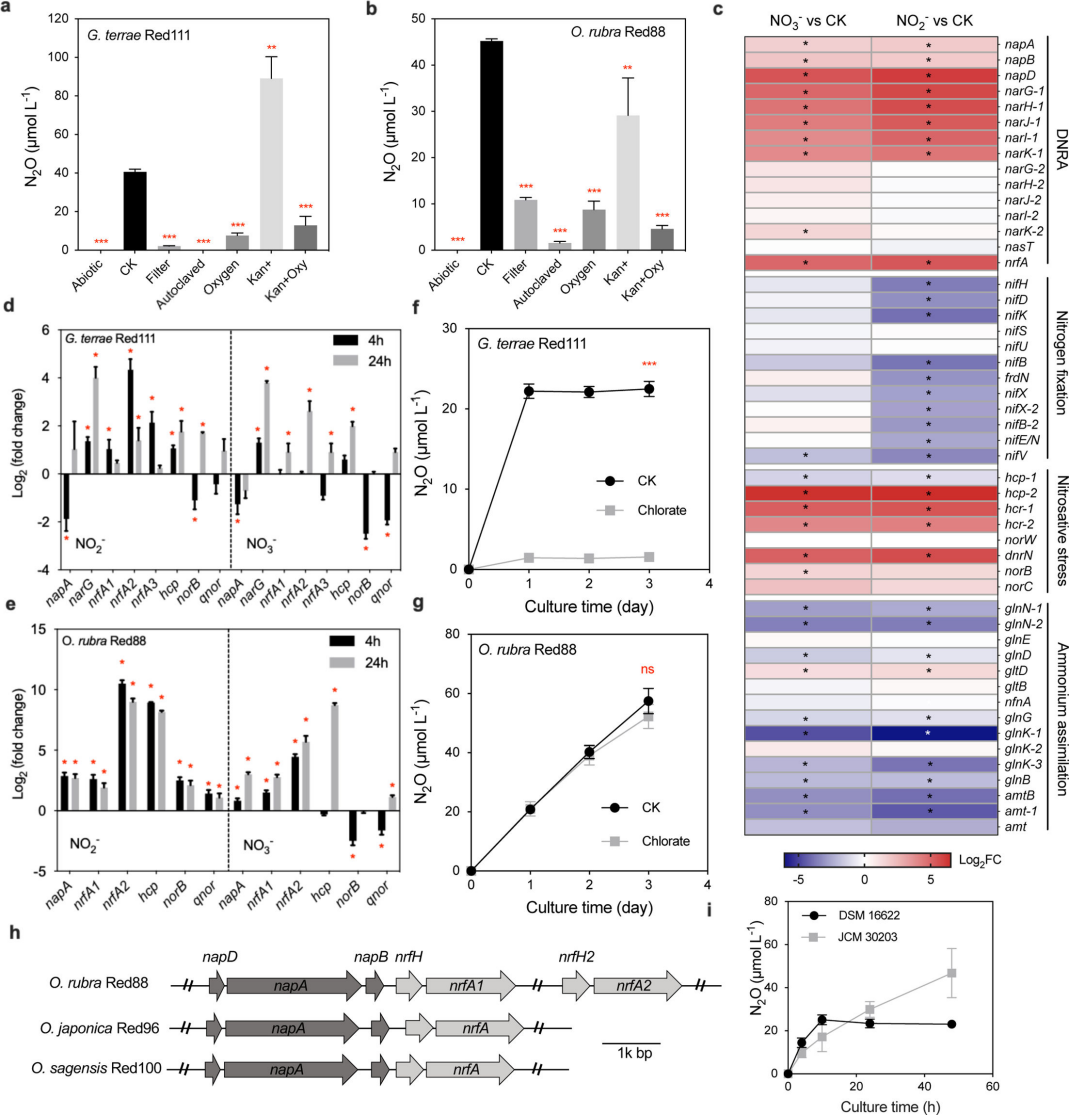
Comparisons of N_2_O productions, gene expressions, and gene structures of *Geobacteraceae* strains. (**a and b**) N_2_O production from the NO_2_^−^ reduction process driven by strains Red111 and Red88 under different treatments: Abiotic, bacteria-free medium; CK, bacteria-containing medium; Filter, filtered bacteria-containing medium; Autoclaved, autoclaved bacteria-containing medium; Oxygen, bacteria-containing medium gassed air; Kan+, bacteria-containing medium supplemented with 100 µg/mL kanamycin; Kan + Oxy, bacteria-containing medium gassed air and supplemented with 100 µg/mL kanamycin. The default medium is NNFM_N. (**c**) The expression levels of nitrogen metabolism related genes from transcriptome analysis in *Geomonas* sp. Red32 with NO_3_^−^ (8 mM, left panel) and NO_2_^−^ (2 mM, right panel) inductions. Red colors represent the upregulated genes, while blue colors represent the downregulated genes. Asterisk indicates significant difference, adjusted *P* < 0.05. (**d and e**) The expression levels of representative genes related to DNRA and nitrosative stress processes from RT-qPCR in strains Red111 and Red88 with NO_3_^−^ (8 mM, right panel) and NO_2_^−^ (2 mM, left panel) inductions for 4 and 24 h. (**f and g**) N_2_O production from the NO_2_^−^ reduction process driven by strains Red111 and Red88 over time with (grey squares) and without (black circles) chlorate additions. (**h**) structure comparison of DNRA-related genes (*nap* and *nrf* clusters) in three *Oryzomonas* strains, Red88, Red96, and Red100. Arrows indicate open reading frames and their orientations in the genomes. (**i**) N_2_O production from the NO_2_^−^ reduction process driven by *nar*/*nap*-absent strains *Geomonas bemidjiensis* DSM 16622 and *Geobacter anodireducens* JCM 30203 at different culture times. Asterisks indicate significant difference, *** *P* < 0.001, **P < 0.01, ns indicates insignificant difference, *P* > 0.05.

To elucidate the enzymatic pathways of nitrate-ammonifying N_2_O production, we cultured *G. terrae* Red111 (containing both Nar and Nap) and *O. rubra* Red88 (containing only Nap). Initially, abiotic sources of N_2_O were excluded, as little or no N_2_O production was observed in bacteria-free and autoclaved cultures (Fig. 5a and b), consistent with the differing SP values in nitrate-ammonifiers from the abiotic reaction (Table 1). In contrast, N_2_O production was observed in filtered cultures, where live bacteria had been removed, but free and partial periplasmic enzymes were present, suggesting the enzymatic reactions responsible for N_2_O production during DNRA (Fig. 5a and b). Growth curves and fluorescence microscopy revealed that kanamycin (100ug/mL) reduced biomass and increased cell mortality of *G. terrae* Red111 and *O. rubra* Red88 in non-concentrated cultures (Fig. S6). Consequently, kanamycin was used to prepare cell lysates. Kanamycin addition significantly increased N_2_O production in *G. terrae* Red111 (*P* < 0.01) but decreased it in *O. rubra* Red88 (*P* < 0.01) (Fig. 5a and b). This increase in *G. terrae* Red111 possibly resulted from the release of more intracellular enzymes, whereas the decrease in *O. rubra* Red88 may be attributed to the cessation of extracellular/periplasmic enzyme synthesis, suggesting different enzyme profiles between these strains, consistent with their varying responses to c-PTIO (Fig. 4b and c). Moreover, kanamycin slightly increased N_2_O production with SNP addition in both strains (*P* > 0.05) (Fig. S5), suggesting the intracellular enzymes in both strains facilitating the reduction of NO to N_2_O. The addition of oxygen significantly decreased N_2_O production in both strains with NO_2_^−^ or SNP (*P* < 0.001, Fig. 5a and b; Fig. S5), implying that an oxygen-sensitive enzyme is responsible for N_2_O production, aligning with the anaerobic metabolism of DNRA.

To identify the key enzymes catalyzing N_2_O production, we quantified the expression levels of genes related to DNRA and nitrosative stress processes under NO_3_^−^, NO_2_^−^, and SNP treatments in *G. terrae* Red111 and *O. rubra* Red88. The target genes were identified based on transcriptomic results from *Geomonas* sp. Red32, which showed high conversion ratios from NO_3_^−^ and NO_2_^−^ to N_2_O (Fig. 3d and h) and was selected for transcriptomic study (Table S2). A total of 1,699 and 1,134 differentially expressed genes were detected in *Geomonas* sp. Red32 under NO_3_^−^ and NO_2_^−^ treatments, respectively (Fig. S7). Genes related to nitrogen fixation and ammonium assimilation were globally downregulated under both NO_3_^−^ and NO_2_^−^ treatments, whereas genes involved in DNRA and nitrosative stress were upregulated (Fig. 5c; Table S3). Given that *Geomonas* sp. Red32 produced N_2_O following NO_3_^−^ or NO_2_^−^ addition (Fig. S8), the upregulated genes in the DNRA and nitrosative stress pathways are connected to N_2_O production.

In *G. terrae* Red111 and *O. rubra* Red88, most genes associated with DNRA and nitrosative stress pathways were significantly upregulated, except for the genes *napA*, *norB*, and *qnor* in *G. terrae* Red111 and *norB* and *qnor* in *O. rubra* Red88, which exhibited low or negative expression levels (Fig. 5d and e; Fig. S9). Denitrifying N_2_O production driven by cNor (encoded by *norBC*) and qNor has been reported with SP values less than 0‰ (23, 45), significantly lower than the SP values observed for DNRA-derived N_2_O. These findings excluded the possibility of cNor and qNor involved in NO reduction in *Geobacteraceae* strains. Interestingly, the *hcp-hcr* gene cluster (encoding the hybrid cluster protein complex, Hcp–Hcr) in the nitrosative stress group showed high expression levels under all conditions (Fig. 5c through e; Fig. S9). Hcp–Hcr is an unusual redox endoenzyme complex with oxygen-sensitive hydroxylamine and NO reductase activity (46), and its function in NO detoxication to N_2_O has been confirmed by Hcp mutants in *Methylobacter* species and *E. coli* (47, 48). Moreover, *hcp-hcr* is the only remaining gene cluster related to NO detoxification present in all *Geobacteraceae* genome sequences (Fig. 2). Given the constant SP values suggesting similar enzymes catalyzing NO to N_2_O in different *Geobacteraceae* strains, we propose the Hcp–Hcr complex as the primary driver of NO reduction to N_2_O in these strains. This finding is distinct from the reported enteric nitrate-ammonifiers that use NO reductase (NorVW) or NO dioxygenase (Hmp or Fhp) for NO detoxication (16, 17).

For the enzyme catalyzing NO_2_^−^ to NO, we first assume Nar in *Geomonas* strains, due to its high expression and central role in the gene co-expression network under the NO_2_^-^ treatment in *Geomonas* sp. Red32 (Fig. 5c and d; Fig. S10). Although Nar is known for catalyzing NO_3_^−^ to NO_2_^−^, its role in catalyzing NO_2_^−^ to NO is less established. To investigate this, we added the Nar inhibitor chlorate (ClO_3_^−^) to the cultures (49). ClO_3_^−^ showed little effect on bacterial growth and NO reduction to N_2_O but completely inhibited NO_3_^−^ reduction in *Geomonas diazotrophica* Red69 (Nar-containing but Nap-absent) and *G. terrae* Red111 (Nar- and Nap-containing), with no effect on Nap-driven *O. rubra* Red88 (Fig. S11 and S12). This indicates the Nar-driven type (Nap deactivated) of *G. terrae* Red111 related to DNRA. ClO_3_^−^ addition further completely inhibited N_2_O production from NO_2_^−^ in *G. terrae* Red111 but had no effect on *O. rubra* Red88 (Fig. 5f and g), underscoring the critical role of Nar in N_2_O production. After numerous unsuccessful attempts to construct genetic mutants of *Geobacteraceae* strains, we moved to use another nitrate-ammonifying strain, *E. coli* MG1655, as an alternative and constructed mutant strains MG1655 ∆*narG* and MG1655 ∆*napA*. Compared with the wild type, strain MG1655 ∆*narG* produced much less N_2_O from NO_2_^−^, while MG1655 ∆*napA* produced similar amounts (Fig. S13). Nar is an oxygen-sensitive membrane-bound enzyme in which the catalytic subunit faces the cytoplasm (50), meeting the proposed characteristics of intracellular enzymes. Thus, Nar is proposed as the enzyme responsible for NO_2_^−^ to NO conversion in Nar-containing *Geomonas* strains.

For Nar*-*deficient *Oryzomonas* strains, Nap and NrfA are the potential candidates for catalyzing NO_2_^−^ to NO, as both are periplasmic enzymes and showed high expression levels in *O. rubra* Red88 under both NO_3_^−^ and NO_2_^−^ treatments (Fig. 5e). To determine the responsible one, we cultured the other two bacteria *Oryzomonas japonica* Red96 and *Oryzomonas sagensis* Red100 and compared their NO_3_^−^ and NO_2_^−^ reduction products. All three strains could produce N_2_O from NO_3_^−^ reduction, but only *O. rubra* Red88 produced N_2_O from NO_2_^−^ reduction (Fig. 3c and g; Fig. S14), indicating that the target enzyme is present in all three strains but in difference from *O. rubra* Red88 to *O. japonica* Red96 and *O. sagensis* Red100. Gene cluster comparisons revealed that the *napDAB* cluster is structurally conserved and shares high sequence similarities (>95%) among the three strains, whereas the *nrfAH* cluster was distinctly different, as *O. rubra* Red88 harbors two paralogs of *nrfAH* (*nrfAH1_Red88_* and *nrfAH2_Red88_*), and *O. japonica* Red96 and *O. sagensis* Red100 harbor only one (*nrfAH1_Red88_* orthologous) (Fig. 5h; Fig. S15). Moreover, the gene *nrfAH2_Red88_* showed higher gene expression than *nrfAH1_Red88_* under NO_3_^−^ and NO_2_^−^ treatments (Fig. 5e), highlighting the difference of NrfA from *O. rubra* Red88 to *O. japonica* Red96 and *O. sagensis* Red100. These comparisons indicate that NrfA is more likely than Nap to be involved in N_2_O production. In addition, two other *Geobacteraceae* strains, *G. bemidjiensis* DSM 16622 and *Geobacter anodireducens* JCM 30203, which lack both *nar* and *nap* genes and are incapable of NO_3_^−^ reduction, also produced N_2_O from NO_2_^−^ reduction with comparable N_2_O ratios and SP values to other studied nitrate-ammonifiers (Table 1; Fig. 5i; Fig. S16). This finding excluded the necessity of Nap in N_2_O production. With these results and the reported crystal structure of NrfA that predicted NO occurrence during NO_2_^−^ reduction to NH_4_^+^ (51), NrfA is supposed as the target enzyme catalyzing NO production in Nap-driven nitrate-ammonifiers.

Although *Geobacteraceae* strains contain one or more copies of the *nrfA* operon, their phenotypes with respect to NO_2_^−^ reduction are not consistent. For example, *Geomonas* sp. Red32, *O. japonica* Red96 and *O. sagensis* Red100 each contain a single copy of *nrfA*, but only *Geomonas* sp. Red32 cannot produce NH_4_^+^ from NO_3_^−^. This variability in NrfA functionality suggests evolutionary divergence among these strains. Phylogenetic analyses based on NrfA sequences reveal that the NrfA proteins of *Geobacteraceae* strains are distributed within Clade I and the outgroup (52), displaying phylogenetic differences at the single species level (Fig. S17). Notably, in *Oryzomonas* strains (Red96, Red100, and Red88-NrfA1), NrfA forms a robust branch within Clade I-I (bootstrap value 0.9) and shares <75% similarity at the amino acid level with other NrfA enzymes. This sequence specificity of NrfA in Clade I-I likely underlies the differential activities observed between *Oryzomonas* and *Geomonas* strains. Most *Geomonas* strains contain both Nar and NrfA enzymes; however, there is currently no evidence supporting the involvement of these Nrf enzymes in N_2_O production. Previous studies have shown that nitrate-ammonifying strains deleting *nrfA* (Nar-containing type) do not influence N_2_O production (16, 17). Given that the Nar complex contains specialized NO_2_^−^/NO_3_^−^ membrane transporters (NarK)(53), we assume that Nar transforms pericellular NO_2_^−^ in high concentration into bacterial cells, thereby alleviating NO_2_^−^ pressure on NrfA proteins and inhibiting NO release from NrfA enzymes in Nar-containing bacteria. Although the enzymatic combinations Nar-Hcp and NrfA-Hcp related to DNRA-derived N_2_O production were functionally identified in *Geobacteraceae* strains, these gene combinations *narG-hcp* and *nrfA-hcp* are widely distributed across the bacterial domain, including within the phyla *Pseudomonadota*, *Bacteroidota*, *Desulfobacterota,* and *Bacillota* (Fig. S18). Thus, this study reveals a previously unrecognized, yet potentially widespread, N₂O production pathway in various bacteria.

### Distinctive isotopocule signatures of DNRA-derived N_2_O with the role in quantifying N_2_O production processes

**Fig 6:**
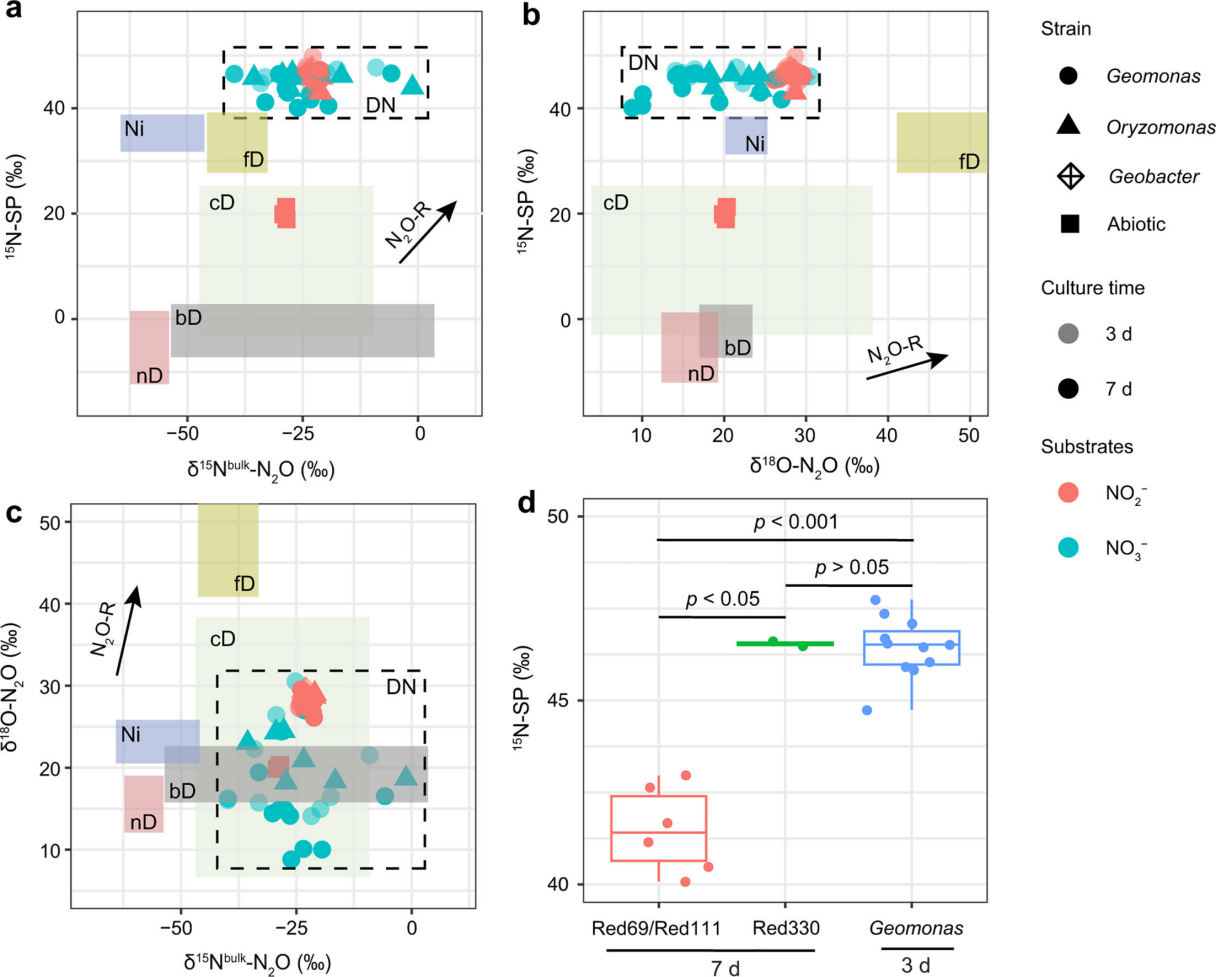
Comparisons of N_2_O isotopocule signatures from different N_2_O production reactions. (**a–c**) SP/δ^15^N^bulk^, SP/δ^18^O, and δ^18^O/ δ^15^N^bulk^ plots showing the isotopocule signatures of N_2_O from various nitrogen metabolism processes. nD, nitrifier denitrification; bD, bacterial denitrification; fD, fungal denitrification; cD, chemical denitrification; NI, nitrification; DN, DNRA. The black arrows denote the N_2_O reduction (N_2_O-R) by bacterial denitrification based on early studies [slope *ε*(SP) / *ε*(δ^15^N) = 0.96, slope *ε*(SP) / *ε*(δ^18^O) = 0.45, slope *ε*(δ^18^O) / *ε*(δ^15^N) = 2.21]. The value ranges (except for DNRA obtained in this study) are retrieved from Ref (9). (**d**) SP value comparisons among strains Red69/Red111 (7 days), strain Red330 (7 days), and all *Geomonas* strains (3 days) with the substrate NO_3_^−^. The raw data are presented in Table S1.

The isotopocule signatures of DNRA-derived N_2_O (except abnormal values from mixed processes) range from 43.0‰ to 49.9‰ for SP, −39.9‰ to −5.8‰ for δ^15^N^bulk^, and 14.1‰ to 30.5‰ for δ^18^O (Table 1; Fig. 6a through c). These values are notably distinctive compared to those from other N_2_O generation processes (9), particularly the SP values, which surpass those reported for any other processes (Fig. 6a and b), suggesting that SP is a robust and independent tool for identifying DNRA-derived N_2_O. In contrast, the δ^15^N^bulk^ and δ^18^O values (isotopic fractionation from substrates) of the produced N_2_O show greater variability (δ^15^N^bulk^ = −24.6 ± 6.0‰, δ^18^O = 23.6 ± 6.3‰) than the SP values and overlap with values from other known N_2_O production processes (Table 1; Fig. 5a through c), indicating their limited effectiveness as indicators for N_2_O partitioning. However, the combined δ^18^O/δ^15^N^bulk^ plot enables clear differentiation of DNRA-related N_2_O from other processes, including nitrification, nitrifying-denitrification, and fungal denitrification (Fig. 6c). This highlights the utility of dual isotope plots in distinguishing N_2_O sources. Previous studies have noted that the N_2_O reduction process mediated by N_2_O reductase (NosZ) can elevate SP values appreciably (54), which may obscure the identification of high SP value processes. Our results show that the slope of SP/δ^15^N^bulk^ for residual N_2_O caused by N_2_O reduction from nitrification and fungal denitrification sources overlaps with the DNRA zone (Fig. 6a), while this slope of SP/δ^18^O does not overlap with the DNRA zone (Fig. 6b). Thus, SP/δ^18^O provides a more precise means of distinguishing DNRA-derived N_2_O from other pathways, offering enhanced resolution compared to SP alone. Indeed, high SP values over 45‰ have been reported in a nitritation-anammox reactor (55), which could be attributed to DNRA-derived N_2_O as characterized in this study.

N_2_O isotopocule signatures also serve as tools for identifying and quantifying mixed N_2_O production processes through isotope mass balance. For instance, the N_2_O production from NO_3_^−^ reduction by *G. terrae* Red111 at 7-day culture suggests mixed processes, given its lower SP values (41.1‰–41.9‰) than others (Fig. 6d). To elucidate the contributing processes, we introduced another two strains, *G. diazotrophica* Red69 and *Geomonas silvestris* Red330, and measured their N_2_O isotopocule signatures. Both *G. terrae* Red111 and *G. diazotrophica* Red69 showed similar decreases in SP value over prolonged culture periods, whereas *G. silvestris* Red330 did not (Table 1), despite all strains showing similar NO_3_^−^/NO_2_^−^ reduction rates (Fig. S18). This discrepancy indicates the differences between *G. silvestris* Red330 and those two strains causing the decrease in SP values. Genetic comparison revealed that *G. diazotrophica* Red69 and *G. terrae* Red111 contain *norBC* clusters, while *G. silvestris* Red330 does not (Fig. 2). cNor has been documented to catalyze NO to N_2_O during denitrification, with known SP values ranging from 7.5‰ to 3.7‰ (9). Given that NO is a precursor to N_2_O in DNRA, the partial involvement of cNor in *G. diazotrophica* Red69 and *G. terrae* Red111 likely contributes to the observed SP value reductions over time. Quantitative analysis of mixed N_2_O production processes revealed that DNRA-related enzymatic reactions account for 93.9% and 94.0% of N_2_O production during NO_3_^−^ reduction at the 7-day culture of *G. diazotrophica* Red69 and *G. terrae* Red111, respectively. This approach has also been applied to quantify four mixed N_2_O^−^ production/reduction processes using N_2_O isotope mass balance (56, 57), displaying its significant potentials for environmental research. However, estimating pathways based on only two or three N_2_O isotopic dimensions can lead to an underdetermined set of equations. Thus, additional measurements, such as microbial ecological data or pathway modelling approaches, are necessary for accurate determination of extra pathways.

Previous studies on pure cultures of nitrifiers and fungal denitrifiers have demonstrated that N_2_O formed from asymmetrical precursors (N-N-O) typically exhibits high SP values (>20‰) owing to sequential binding mechanisms, whereas low SP values around 0‰ occurring in bacterial denitrification involve dimerization of NO (23, 58). The sequential reaction of asymmetrical precursors is thus supposed to account for the synthesis of DNRA-derived N_2_O molecules. Although the mechanism for the high SP values in DNRA-derived N_2_O is not yet fully elucidated, we hypothesize that specific enzyme activities or catalytic centers govern the sequential binding of NO, resulting in distinct fractionations during the addition of the first or second NO precursor. Further studies involving enzymatic structures is needed to bridge this gap. Coincidentally, the predicted equilibrium SP value of N_2_O at room temperature was reported as 45‰ based on theoretical modeling (59), comparable to that of DNRA-derived N_2_O. We thus believe that the high SP values of DNRA-derived N_2_O may provide novel insight into N_2_O formation mechanisms in follow-up studies.

**Fig 7:**
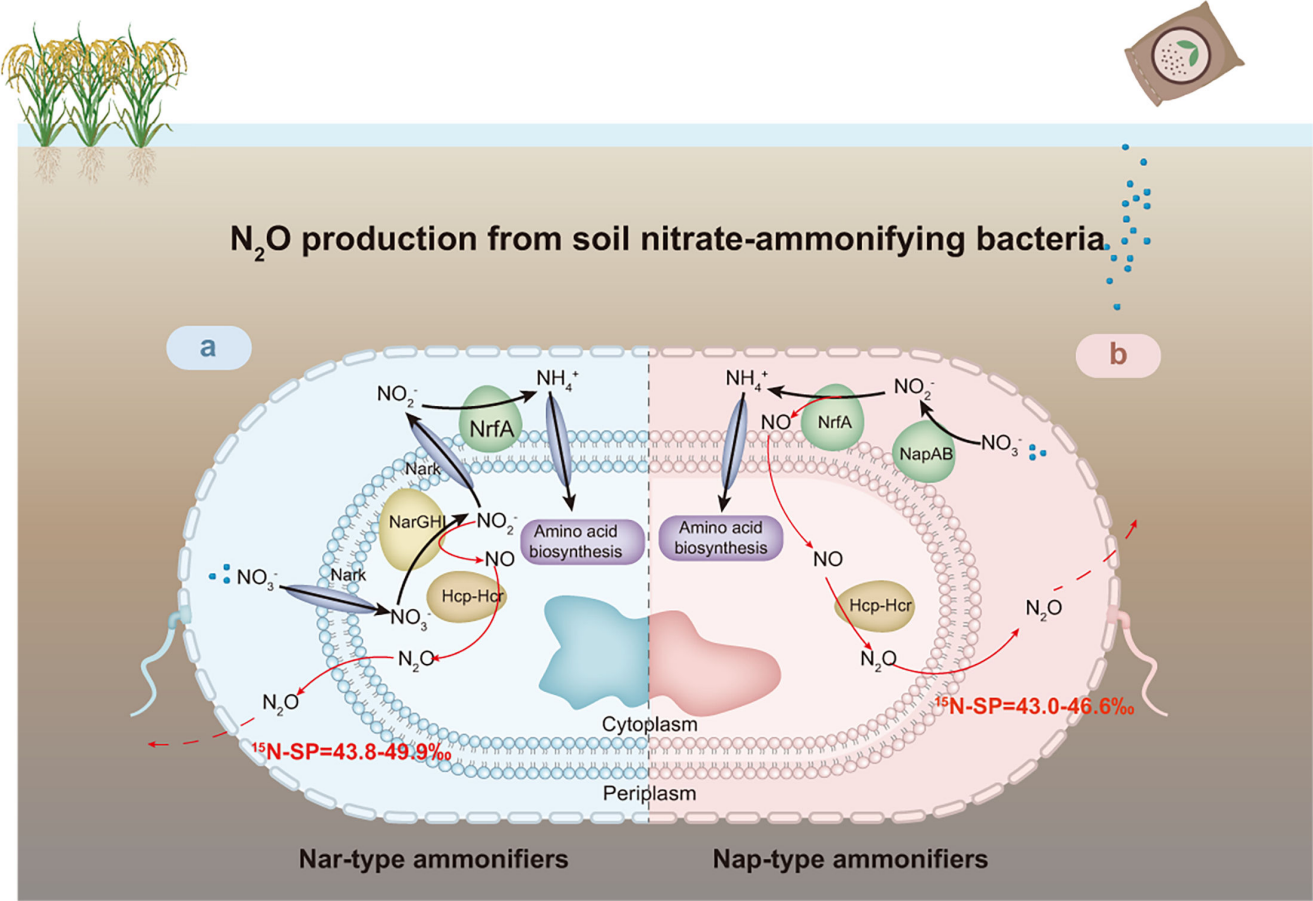
Schematic description of DNRA-derived N_2_O production. (**a**) N_2_O production pathway in Nar-driven *Geomonas* strains. (**b**) N_2_O production pathway in Nap-driven *Oryzomonas* strains. Red arrows with relative thin lines indicate the N_2_O production as a byproduct from NO_2_^−^ during the DNRA process. Key enzymes are indicated as nitrate/nitrite antiporter (NarK), respiratory nitrate reductase (NarGHI), periplasmic nitrate reductase (NapAB), cytochrome *c* nitrite reductase (NrfA), and hybrid cluster protein complex (Hcp–Hcr). The enzymes in different colors denote different enzyme types, green labels the exoenzyme (Nap) or the outer membrane protein (Nrf), brown labels the endoenzyme (NarGHI and Hcp–Hcr complex), and blue labels the membrane transport proteins (NarK and others). The shown ranges of SP values were summarized from the NO_3_^−^/NO_2_^−^ reduction process driven by *Geomonas* or *Oryzomonas* strains in this study.

Our results elucidate the N_2_O production pathways (NO_2_^−^-NO-N_2_O) in both Nar- and Nap-type DNRA bacteria within the *Geobacteraceae* family. We identified two novel enzymatic pathways for N_2_O generation in nitrate-ammonifiers: 1) Nar and the Hcp–Hcr complex catalyzed the NO_2_^−^-NO-N_2_O process in Nar-type nitrate-ammonifiers; 2) NrfA and the Hcp–Hcr complex catalyzed the NO_2_^−^-NO-N_2_O process in Nap-type nitrate-ammonifiers (Fig. 7). Furthermore, DNRA-derived N_2_O exhibits the highest SP values reported to date across various N_2_O production pathways in pure culture experiments. Cross plots of SP with δ^15^N^bulk^ and δ^18^O values clearly distinguish DNRA-derived N_2_O from other known production pathways. These results are crucial for precisely modeling global greenhouse emissions. Nevertheless, the limited nitrate-ammonifiers in one group of *Geobacteraceae* are insufficient to obtain a comprehensive understanding of DNRA-derived N_2_O production, as environmental nitrate-ammonifiers exhibit a range of enzyme combinations related to N metabolism. On this basis, large-scale nitrate-ammonifier screening with N_2_O production pathway parsing will be considered in future studies.

## MATERIALS AND METHODS

### Bacterial strains and cultivation

Nine bacterial strains of the family *Geobacteraceae* and a reference strain *E. coli* K-12 MG1655 were cultured for this study. Among these, *Geomonas* sp. Red32 was recently isolated from paddy soils and has been deposited in the Japan Collection of Microorganisms (JCM 33031) and Marine Culture Collection of China (MCCC 1K03692). *G. bemidjiensis* DSM 16622 was obtained from the Deutsche Sammlung von Mikroorganismen und Zellkulturen (Braunschweig, Germany), while *Geobacter anodireducens* JCM 30203 was sourced from the Japan Collection of Microorganisms (Tsukuba, Japan). Others, including three *Geomonas* strains Red69, Red111, and Red330 and three *Oryzomonas* strains Red88, Red96, and Red100, were described in our previous works (30–32, 34) and reactivated for this study. *E. coli* K-12 MG1655 was acquired from the National BioResource Project (NBRP)-*E. coli* (Mishima, Japan). Gene knockout mutants of *E. coli* K-12 MG1655 were constructed as described previously (60) and hereafter termed as MG1655 ∆*nar* and MG1655 ∆*nap*, representing the markerless *narG* and *napA* deletion mutants, respectively.

Unless otherwise specified, the nine *Geobacteraceae* strains were routinely cultured in 50 mL glass serum bottles containing 20 mL of anoxic nitrogen-free freshwater medium (NFFM, pH 6.8) supplemented with 10 mM acetate as the electron donor and carbon source and 10 mM fumarate (NFFM_F) or nitrate (NFFM_N) as the electron acceptor. Cultures were incubated at 30°C without shaking. The composition of NFFM was consistent with that described previously (34), except that NH_4_Cl was omitted due to the N_2_-fixing capability of these strains. The only non-N_2_-fixing strain, *Geob. anodireducens* JCM 30203, was cultured equally with the addition of 5 mM NH_4_Cl to the medium. Serum bottles containing NFFM were neck-sealed with butyl-rubber stoppers and aluminum crimps, with the gaseous phase replaced by N_2_/CO_2_ (80:20, v/v). *E. coli* K-12 MG1655 and its mutants were routinely cultured using aerobic LB broth (Nihon Pharmaceutical, Japan) at 37°C with shaking at 120 rpm and anaerobically cultured when monitoring NO_2_^−^ reduction using LB broth supplemented with 2 mM NO_2_^−^ .

### Measurement of NO_3_^−^ and NO_2_^−^ reduction to ammonium

NO_3_^−^ reduction experiments were conducted using the strains cultured in NNFM_N, while NO_2_^−^ reduction experiments involved culturing the strains in NNFM_F for 2 days until the biomass reached an optical density of >0.02 (OD_600_) and then 2 mM NaNO_2_ was injected into the bottles. The sampling was started from the time of NaNO_2_ addition. For sample collection, 1 mL of culture was withdrawn from each bottle at designated time intervals, and the bacterial biomass was measured at OD_600_. After that, the samples were filtered through 0.22 µm syringe filters prior to preserved in a freezer (−20°C) for subsequent measurement. The concentrations of NO_3_^−^ and NO_2_^−^ were measured using an ion chromatograph (Dionex ICS-900) equipped with a Dionex IonPac AS12A analytical column (Thermo Fisher Scientific, USA). NH_4_^+^ concentrations were determined using the indophenol-blue method followed by colorimetry (61). The produced N_2_O in the headspace gas of the cultural bottles (1 mL) was measured using a gas chromatograph equipped with a Porapack Q column and an electron capture detector (GC-ECD, GC-2014, Shimadzu, Japan). The temperature of the injector, detector, and column were 90°C, 345°C, and 80°C, respectively, with an Ar/CH_4_ carrier gas flow rate of 150 mL/min. The N_2_O dissolved in the liquid medium was calculated using the Bunsen absorption coefficient (*α* = 0.544, 25°C)(62). The effect of carbon-to-nitrate (C:N) ratios on DNRA activities and bacterial growth was investigated using *G. terrae* Red111 in NNFM_N for 1 week. Different C:N ratios were prepared using a constant NO_3_^−^ concentration of 10 mM as the nitrogen source and varying acetate concentrations (5–40 mM) as the carbon source.

### Control experiments to explore N_2_O production pathways

To assess the contribution of NO to N_2_O, a NO scavenger, 2-(4-carboxyphenyl)−4,4,5,5-tetramethylimidazoline-1-oxyl-3-oxide (c-PTIO, Santa Cruz Biotechnology, USA), was applied during bacterial culture in NNFM_N. The effect of c-PTIO on bacterial growth was evaluated using *G. terrae* Red111 at various concentrations (0, 50, 100, and 300 µM). NO inhibition tests were performed using 100 µM c-PTIO and quantified by the comparison of N_2_O concentration with c-PTIO-free cultures. L-N^G^-monomethyl arginine acetate (L-NMMA, Dojindo, Japan), used to investigate the role of endogenous NO in N_2_O production, was applied at a work concentration of 100 µM. Hydroxylamine concentrations were measured as described previously (63). The effect of kanamycin (100 μg/mL) on bacterial growth was monitored using growth curves and cell viability assays, with the latter performed *via* fluorescence microscopy (Carl Zeiss Axio Scope A1, Germany) following bacterial staining with a live/dead cell viability assay kit (Bestbio, China). For treatments under filtered, autoclaved, oxygen-containing and kanamycin-supplemented conditions, cultures were prepared using NNFM_N inoculated with target strains, and then dispensed into 30 serum vials (5 mL culture in 20 mL vials, five replicates/treatment) of each strain once the biomass reached an OD_600_ >0.02. Filtered cultures were treated using 0.22 µm syringe filters, autoclaved cultures were prepared by autoclaving at 121°C for 20 min, and kanamycin-supplemented cultures were prepared by injecting kanamycin solution to a final concentration of 100 µg/mL. The gaseous phase in the vials was then replaced with N_2_/CO_2_ (80:20, v/v), except for the oxygen-containing cultures. After that, 2 mM NO_2_^−^ or 80 µM sodium nitroprusside (SNP, Sigma, USA; NO donor) was injected into sub-cultures or cell-free media as required, which were then cultured at 30 °C for 3 days before N_2_O sampling. The effect of sodium chlorate (NaClO_3_; 20 mM) on Nar, Nap, and both Nar and Nap containing strains, *G. diazotrophica* Red69, *O. rubra* Red88, and *G. terrae* Red111, respectively, was examined using NNFM_N and NNFM_F supplemented with 2 mM NO_2_^−^, the consumed and produced N compounds were measured as described above. ClO_3_^−^ concentrations were determined using an ion chromatograph as described above.

### Isotopic analysis of N_2_O, NO_3_^−^, and NO_2_^−^

The N_2_O isotopocule ratios were measured using an automated system containing an autosampler (GX-222 XL liquid handler; Gilson, USA), a customized purge and trap system, a gas chromatographs (6850; Agilent Technologies, USA) equippled with Pola PLOT column, and an isotope ratio mass spectrometer (IRMS, MAT 253, Thermo Fisher Scientific, Germany) (64). High-purity N_2_O (Showa Denko Co., Ltd., purity >99.999%), which was calibrated previously with respect to the international isotopic standards air-N_2_ for δ^15^N and Vienna standard mean ocean water (VSMOW) for δ^18^O, were diluted with high-purity N_2_ and measured parallelly to correct and calibrate the ratios. Values of δ^15^N^bulk^, δ^15^N^α^, and δ^18^O were calculated as described previously (65). Values of δ^15^N^β^ and SP were obtained as follows: δ^15^N^β^ =2δ^15^N^bulk^ - δ^15^N^α^; SP = δ^15^N^α^ - δ^15^N^β^. The typical analytical precisions are 0.2‰ for δ^15^N^α^ and 0.1‰ for δ^15^N^bulk^ and δ^18^O. Abiotic control N_2_O samples were obtained from bacteria-free NNFM supplemented with 2 mM NaNO_2_, acidified to pH <2 using 6M HCl.

The δ^15^N_NO3-_ and δ^18^O_NO3-_ values of the substrate NO_3_^−^ in the medium were measured by the denitrifier method, where NO_3_^−^ was converted into N_2_O by a denitrifying bacterium *Pseudomonas aureofaciens* ATCC 13985. The δ^15^N_NO2-_ and δ^18^O_NO2-_ values of the substrate NO_2_^−^ in the medium were measured by chemical conversion of NO_2_^−^ into N_2_O using the azide method. Detailed descriptions of these approaches and the procedures used for calibration of multiple standards (NO_3_^−^, NO_2_^−^, and N_2_O) for isotopomer ratios are indicated in Ref (66). The isotopologues of the substrate NO_3_^−^ were determined as δ^15^N_NO3-_ of 2.4 ± 0.1‰ and δ^18^O_NO3-_ of 17.9 ± 0.3‰, while those of the substrate NO_2_^−^ were δ^15^N_NO2-_ of −1.8 ± 0.3‰ and δ^18^O_NO2-_ of 3.7 ± 0.2‰. These isotopologues of the substrates were then used for correcting the absolute δ^15^N^bulk^ and δ^18^O values of N_2_O.

To clarify the mixed N_2_O production processes during NO_3_^−^ reduction by strains Red69 and Red111, a quantitative estimation was performed using isotope mass balance. The fractional contributions (*f* value) to total N_2_O production based on SP were expressed as:


$$SP_{7-day}=SP_{3-day}\times f_{DNRA}+SP_{cNor}\times (1-f_{DNRA})$$

where SP_7-day_ and SP_3-day_ represent the measured SP values of strains Red69 and Red111 in NO_3_^−^ reduction at the culture time of 7 and 3 days, respectively. SP_cNor_ denotes the SP values of denitrifying N_2_O produced from cNor (−5.9 ± 2.1‰)(67), and *f_DNRA_* indicates the relative contribution of DNRA-mediated process.

### Phylogenomic analysis and comparative genomics

The reference genome sequences utilized in this study were retrieved from the NCBI database with accession numbers provided in Table S4. The phylogenomic tree was constructed using the up-to-date bacterial core gene set (UBCG) pipelines equipped with RAxML tool based on the amino acid sequences of 92 concatenated core genes (35). The presence and absence of functional genes related to the N metabolism in *Geobacterales* strains were determined through genome annotations by BlastKOALA server of the KEGG database (68) and RAST server (ClassicRAST annotation scheme) of the SEED database (69). In cases of inconsistencies between the two annotation servers, manual examination was performed using BLASTp against the KEGG GENES and NCBI-nr databases. For NrfA-based phylogenetic tree construction, amino acid sequences were retrieved from sequenced genomes available in the NCBI database, following the guidance in Ref (52, 70). MEGA X (71) and IQ-TREE (72) were used to align the sequences and generate the trees, respectively. AnnoTree v1.2, a tool used for exploration of functional genes across microbial tree of life, was used to search *narG-hcp* and *nrfA-hcp* gene combinations across the bacterial domain (Table S5)(73). All phylogenetic trees were polished by the interactive tree of life (iTOL) v5 (74).

### Transcriptome analysis and real-time quantitative PCR

*Geomonas* sp. Red32 was inoculated into nine serum bottles (5% inoculum size, v/v) containing 20 mL NFFM_F and cultured for 2 days at 30°C. Then, NO_3_^−^ and NO_2_^−^ stock solutions were individually injected to three of the serum bottles to a final concentration of 10 and 2 mM, respectively. The remaining three serum bottles supplemented with the same amount of autoclaved water were the control group. After a further 4-h incubation, biomass was harvested by centrifugation at 10,000×*g* for 10 min at 4°C and then frozen in liquid nitrogen prior to storage in a freezer at −80°C. Total RNA was extracted using the total RNA extraction kit (Isogen II, Nippon Gene, Japan) and subsequently sent to Novogene Co. Ltd. (Beijing, China) for transcriptome sequencing on the Illumina NovaSeq 6000 platform. Differential gene expression analysis was performed using Bowtie2 (75) and DESeq2 (version 1.24.0) (76). Gene expression was quantified using the transcripts per million (TPM) method. Differentially regulated genes were defined with the criteria of a more than twofold change in expression and a false discovery rate-adjusted *P* < 0.05. Co-expressional networks were conducted by Cytoscape (version 3.8.2)(77) based on the Pearson correlation coefficient (Pearson’s r) calculated using R (version 4.0.4).

cDNA was synthesized using the ReverTra AceTM qPCR RT Master Mix with gDNA Remover (Toyobo, Japan). Real-time quantitative PCR (RT-qPCR) was performed using the StepOnePlus Real-Time PCR System (Applied Biosystems, USA) using a 20 µL reaction mixture containing 10 µL of PowerUp SYBR Green PCR Master Mix (2Χ, Thermo fisher), 1 µL of the primer (10 μΜ), 1 µL of the cDNA, and 8 µL of distilled water. The reaction condition was the default program of the instrument with the Fast-cycling model. The primer pairs for RT-qPCR were listed in Table S6. Fold changes of gene expression were calculated by comparative C_T_ method. The reference gene *rpoB* was used to normalize the expression levels of target genes in this study (reference gene selection see Supplementary method). The consistency assessment of transcriptional and RT-qPCR results was described in the Supplementary method.

### Statistical analyses

Statistical analyses and graphical visualizations were performed using the computing environment R v4.0.4 and GraphPad Prism v8.0.2. Significant differences (*P-* values) in N_2_O/NH_4_^+^ productions, bacterial biomass, and chlorate consumptions were assessed using the Student’s *t*-test for comparisons between two groups, or one-way ANOVA followed by LSD significant difference test for comparisons involving more than two groups. For RT-qPCR results and N_2_O isotopocule signatures, statistical significance was determined using the Mann–Whitney U tests for two-group comparisons or Kruskal–Wallis H tests for comparisons involving more than two groups.

## Data Availability

The transcriptomic data generated in this study are publicly available in the NCBI under the BioProject accession number PRJNA801072.

## SUPPLEMENTAL MATERIAL

Supplemental Material mbio.02540-24-s0001.pdf Supplemental methods; Figures S1 to S21.

Table S1 mbio.02540-24-s0002.xlsx Detailed isotopocule signatures of N_2_O produced from NO_3_^-^ and NO_2_^-^ reduction processes by *Geobacterales* strains and abiotic reactions.

Table S2 mbio.02540-24-s0003.xlsx Summary statistics of transcriptomic data and mapping results.

Table S3 mbio.02540-24-s0004.xlsx Detailed transcriptomic data focus on the nitrogen metabolism process of strain Red32 under NO_3_^-^ and NO_2_^-^ treatments.

Table S4 mbio.02540-24-s0005.xlsx Detailed information of species in the family *Geobacteraceae*.

Table S5 mbio.02540-24-s0006.xlsx Hit numbers and hit ratios of *narG-hcp* and *nrfA-hcp* gene combinations in the Bacteria domain.

Table S6 mbio.02540-24-s0007.xlsx Primers used in this study.

ASM does not own the copyrights to Supplemental Material that may be linked to, or accessed through, an article. The authors have granted ASM a non-exclusive, world-wide license to publish the Supplemental Material files. Please contact the corresponding author directly for reuse.
