# Supplementary material for: Unprecedented N2O production by nitrate-ammonifying Geobacteraceae with distinctive N2O isotopocule signatures
Source: mBio. 2024 Oct 30;15(12):e02540-24. doi: 10.1128/mbio.02540-24 (PMC11633192; doi:10.1128/mbio.02540-24)
Supplement: Supplemental Material — Supplemental methods; Figures S1 to S21. [file mbio.02540-24-s0001.pdf]

Supplemental Material

Unprecedented N_2O production by nitrate-ammonifying *Geobacteraceae* with distinctive N_2O isotopocule signatures

Zhenxing Xu[1,2*], Shohei Hattori[3,4], Yoko Masuda[2,5*], Sakae Toyoda[6], Keisuke Koba[7], Pei Yu[8], Naohiro Yoshida[9,10], Zong-Jun Du[1], Keishi Senoo[2,5]

1. Marine College, Shandong University, Weihai, China
2. Department of Applied Biological Chemistry, Graduate School of Agricultural and Life Sciences, The University of Tokyo, Tokyo, Japan
3. International Center for Isotope Effects Research (ICIER), Nanjing University, Nanjing, China
4. Frontiers Science Center for Critical Earth Material Cycling, State Key Laboratory for Mineral Deposits Research, School of Earth Sciences and Engineering, Nanjing University, Nanjing, China
5. Collaborative Research Institute for Innovative Microbiology, The University of Tokyo, Tokyo, Japan
6. School of Materials and Chemical Technology, Institute of Science Tokyo, Yokohama, Japan
7. Center for Ecological Research, Kyoto University, Shiga, Japan
8. SDU-ANU Joint Science College, Shandong University, Weihai, China
9. Earth-Life Science Institute, Institute of Science Tokyo, Tokyo, Japan
10. National Institute of Information and Communications Technology, Tokyo, Japan

Author for correspondence:
Zhenxing Xu, Ph.D.
Email: xuzx.ut@gmail.com; xuzhenxing@sdu.edu.cn
Yoko Masuda, Ph.D.
Email: ygigico@gmail.com

ZX ORCID#: 0000-0002-6210-5348
YM ORCID#: 0000-0002-2233-8497

This file include:

Methods: Page 2-3
Reference: Page 4
Figures: Page 5-26 (Fig. S1 to Fig. S21)

Supplementary Method

Reference gene selection in RT-qPCR

The quantitative analysis of gene expression using RT-qPCR adopted the comparative C_T method (also referred as $2^{-\Delta\Delta Ct}$ method) in this study, the reference genes with stable expression levels under different conditions are necessary for data normalization. To achieve this purpose, seven candidate housekeeping genes, *rpoB*, *rpoD*, *gyrB*, *recC*, *recA*, *proC* and 16S rRNA gene, which were reported to suit transcript normalization in bacterial strains close to the order *Geobacterales*(1–4), were assayed parallelly using the strain *Geomonas terrae* Red111. The different conditions as treatments adopted in this study were used different electron acceptors, i.e., fumarate (10 mM), nitrate (10 mM) and iron(III)-NTA (5 mM) in the anoxic nitrogen-free freshwater medium and different culture times, i.e., 2 day and 7 day. After bacterial biomass were collected, RNA extraction and cDNA synthesis were performed using the total RNA extraction kit (Isogen II, Nippon Gene, Japan) and the ReverTra AceTM qPCR RT Master Mix with gDNA Remover (Toyobo, Japan), respectively. The cDNA concertation was measured using Qubit 2.0 fluorometer (Invitrogen, MA, USA) with the corresponding reagents and was then diluted to similar concentrations for all samples using UltraPure Distilled Water (Invitrogen, MA, USA). RT-qPCR was performed using the same reaction systems as described in the main content with the special primers listed in Table S6. The expression stabilities of these candidate reference genes were quantified and ranked using four statistical tools, geNorm(5), NormFinder(6), BestKeeper(7), and the comparative ΔCt method(8), and a web-based analysis tool, RefFinder(9). Among them, raw Ct (quantification cycle) values were directly used for calculation of BestKeeper and RefFinder tools, while for geNorm and Normfinder tools, raw Ct values were transformed into relative quantities (RQ), using the formula $RQ = E^{-\Delta Ct}$, where E is the amplification efficiency (default value 2, responding to 100% amplification efficiency), ΔCt is calculated using the formula $\Delta Ct = Ct_{min} - Ct_{sample}$, with Ct_{sample} being the raw Ct value for each gene, and Ct_{min} being the minimal raw Ct value (the sample with the highest relative quantity) over a range of samples(10).

The four statistical tools showed different rankings of the seven candidate genes (Fig. S20a-d). For example, the gene *rpoB* is ranked as the most stable gene based on the comparative ΔCt

method, while *recC* is the most stable one based on the Bestkeeper method. To get an overall ranking of these candidate housekeeping genes, a comprehensive ranking tool, namely RefFinder, was used for a final evaluation, as it could assign an appropriate weight to an individual gene and calculate the geometric mean of their weights for the overall final ranking based on the rankings from other four statistical tools(9). The final ranking from the most stable gene to the least stable gene was determined in order as *rpoB*, *rpoD*, *gyrB*, *recC*, *recA*, *proC* and 16S rRNA gene (Fig. S20e). Thus, *rpoB* is the most stable one and potentially to be the reference gene for the following data normalization. Moreover, the quality of special primers for *rpoB* gene amplification in the *Geomonas* strain Red111 was also assayed (Fig. S20f,g). A single peak on the melt curve confirmed the specificity of the amplicon. The efficiency curve using a pool of all the cDNA samples was carried out to calculate the amplification efficiency and the correlation coefficient (R^2) of the target primer pair. The amplification efficiency was determined as 100.28% with R^2 of 0.9955, which is within the acceptable range for a reliable real-time PCR quantification. Altogether, *rpoB* gene with its primer pair used in this study was the optimum for normalizing RT-qPCR analysis in *Geobacteraceae* strains.

Consistency assessment of transcriptional and RT-qPCR results

The relative gene expression levels obtained from the transcriptional analysis and RT-qPCR showed a consistent variation trend that the up/down-regulated genes in the transcriptional analysis also showed an identical change in the RT-qPCR results (Fig. S21a,b). Moreover, the linear fitting tests revealed that the relative gene expression levels of the two methods showed strong linear relations with R^2 of 0.8655 and 0.9701 (Fig. S21c,d). These results confirmed that the gene expression levels from the RT-qPCR results were consistent with the transcriptional analysis, thus the gene expression quantified by RT-qPCR with the comparative C_T method and normalized by *ropB* is adoptable in this study.

Reference

1. Li X, McInerney MJ, Stahl DA, Krumholz LR. 2011. Metabolism of H_2 by *Desulfovibrio alaskensis* G20 during syntrophic growth on lactate. Microbiology 157:2912–2921.
2. Mouser PJ, Holmes DE, Perpetua LA, DiDonato R, Postier B, Liu A, Lovley DR. 2009. Quantifying expression of *Geobacter* spp. oxidative stress genes in pure culture and during *in situ* uranium bioremediation. ISME J 3:454–465.
3. Rocha DJP, Santos CS, Pacheco LGC. 2015. Bacterial reference genes for gene expression studies by RT-qPCR: survey and analysis. Antonie Van Leeuwenhoek 108:685–693.
4. Shrestha PM, Rotaru AE, Summers ZM, Shrestha M, Liu F, Lovley DR. 2013. Transcriptomic and genetic analysis of direct interspecies electron transfer. Appl Environ Microbiol 79:2397–2404.
5. Vandesompele J, De Preter K, Pattyn F, Poppe B, Van Roy N, De Paepe A, Speleman F. 2002. Accurate normalization of real-time quantitative RT-PCR data by geometric averaging of multiple internal control genes. Genome Biol 3:research0034.1.
6. Andersen CL, Jensen JL, Ørntoft TF. 2004. Normalization of real-time quantitative reverse transcription-PCR data: A model-based variance estimation approach to identify genes suited for normalization, applied to bladder and colon cancer data sets. Cancer Res 64:5245–5250.
7. Pfaffl MW, Tichopad A, Prgomet C, Neuvians TP. 2004. Determination of stable housekeeping genes, differentially regulated target genes and sample integrity: BestKeeper – Excel-based tool using pair-wise correlations. Biotechnol Lett 26:509–515.
8. Silver N, Best S, Jiang J, Thein SL. 2006. Selection of housekeeping genes for gene expression studies in human reticulocytes using real-time PCR. BMC Mol Biol 7:33.
9. Xie F, Xiao P, Chen D, Xu L, Zhang B. 2012. miRDeepFinder: a miRNA analysis tool for deep sequencing of plant small RNAs. Plant Mol Biol 80: 75–84.
10. Köhsler M, Leitsch D, Müller N, Walochnik J. 2020. Validation of reference genes for the normalization of RT-qPCR gene expression in *Acanthamoeba* spp. Sci Rep 10:10362.
11. Welsh A, Chee-Sanford JC, Connor LM, Löffler FE, Sanford RA. 2014. Refined NrfA phylogeny improves PCR-based *nrfA* gene detection. Appl Environ Microbiol 80:2110–2119.
12. Campeciño J, Lagishetty S, Wawrzak Z, Alfaro VS, Lehnert N, Reguera G, Hu J, Hegg EL. 2020. Cytochrome *c* nitrite reductase from the bacterium *Geobacter lovleyi* represents a new NrfA subclass. J Biol Chem 295:11455–11465.

Supplementary Figures

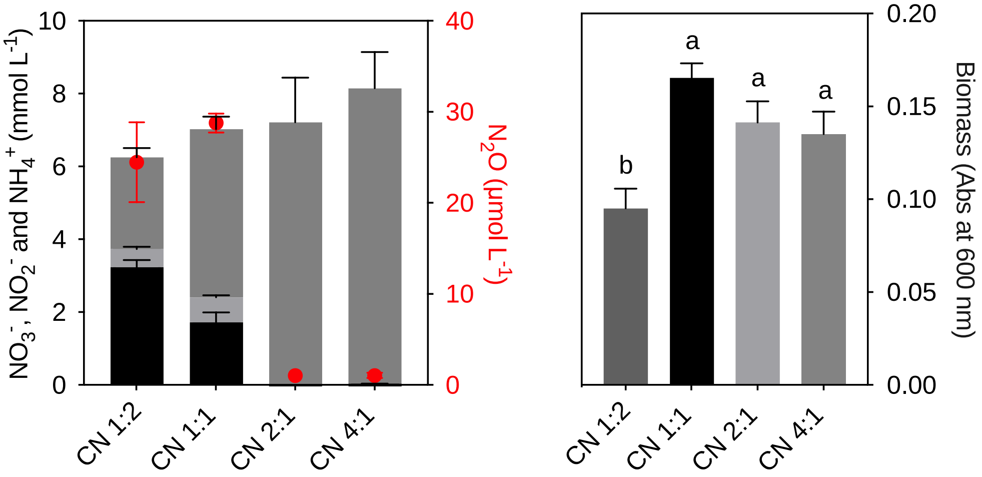

Fig. S1 The effect of C:N ratios on DNRA activities and bacterial growth of *Geomonas terrae* Red111 in NNFM_N for one week. Left panel of every subFig. indicates the concentrations of N composition, including NO_3^- (black), NO_2^- (light grey), NH_4^+ (dark grey), and N_2O (red dots), while right panel indicates the bacterial biomass quantified by the OD_{600} values. The different C:N ratios were prepared using certain NO_3^- (10 mM) as the nitrogen source and different amounts of acetate (5-40 mM) as the carbon source. Different letters on the bars and dots indicate significant differences ($p < 0.05$) among different treatments. All values are shown as mean ± standard deviation (SD).

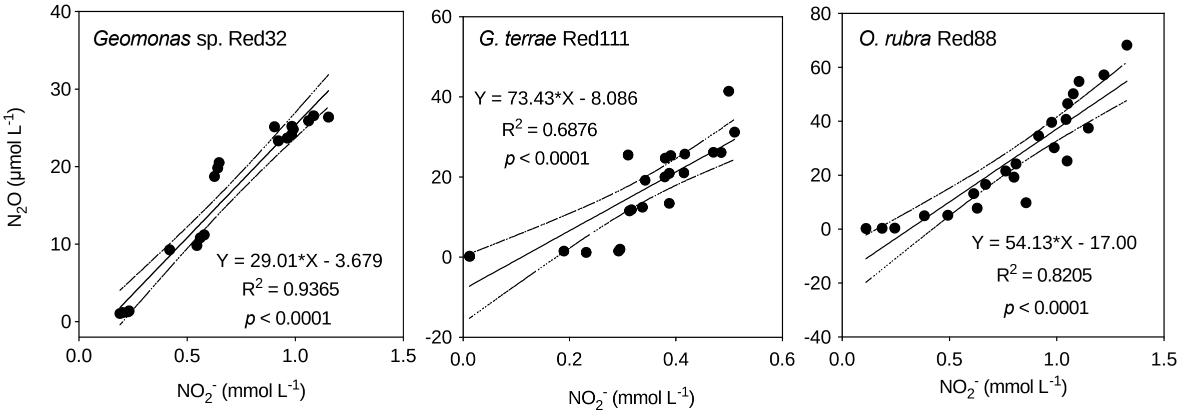

Fig. S2 Relationship between N_2O and NO_2^- concentrations in the cultures during the NO_3^- reduction process driving by different *Geomonas* (Red32 and Red111) and *Oryzomonas* (Red88) strains. The full line indicates the best-fit line based on the simple linear regression model, the range between the two dotted lines indicate the 95% confidence bands of the best-fit line.

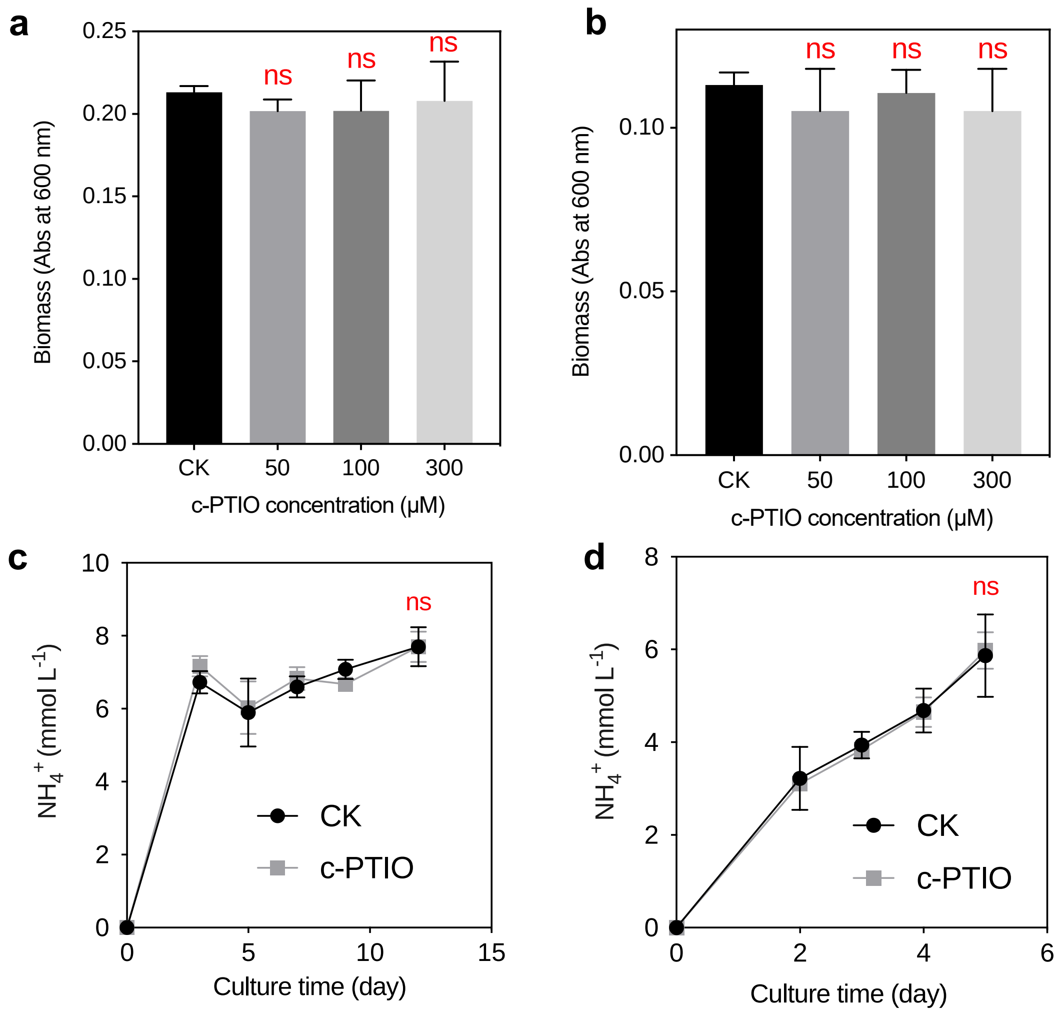

Fig. S3 Biomass and NH_4^+ production curves of strains *Geomonas terrae* Red111 and *Oryzomonas rubra* Red88 during NO_3^- reduction with or without c-PTIO in the medium. a-b, biomass of strains Red111 (**a**) and Red88 (**b**) during NO_3^- reduction with different concentrations of c-PTIO in the medium. c-d, NH_4^+ production curves of strains Red111 (**c**) and Red88 (**d**) during NO_3^- reduction with or without c-PTIO (100 μM) in the medium. The bacterial biomass was represented by the OD values at 600 nm after cultured for 7 days. CK indicates the negative control without c-PTIO addition. ns indicates insignificant difference, $p > 0.05$. The values are shown as mean ± standard deviation (SD).

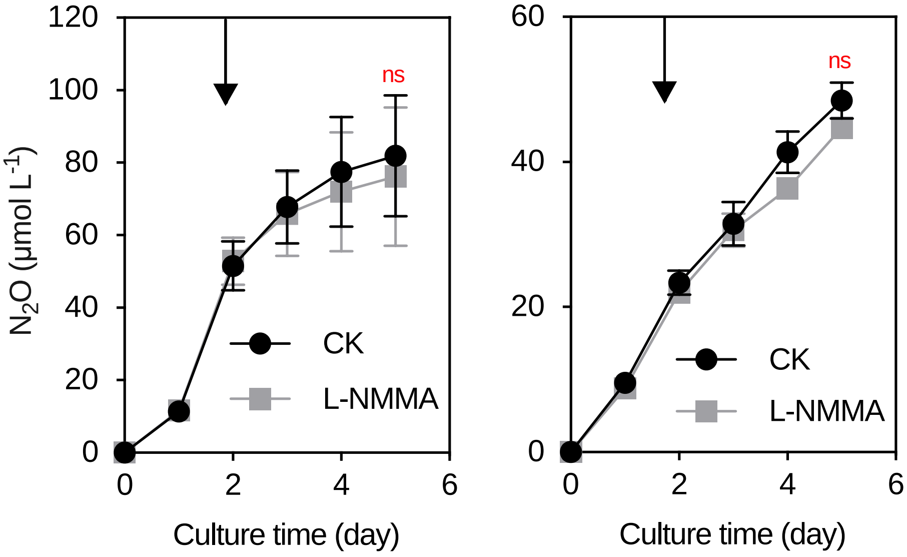

Fig. S4 N_2O production from the NO_3^- reduction process driven by *G. terrae* Red111 (left) and *O. rubra* Red88 (right) at different culture time with (grey squares) and without (black circles) L-NMMA additions. CK indicates control experiments, ns indicates insignificant difference, $p > 0.05$. Arrows indicate the time when L-NMMA was added to the medium. The values are shown as mean ± standard deviation (SD).

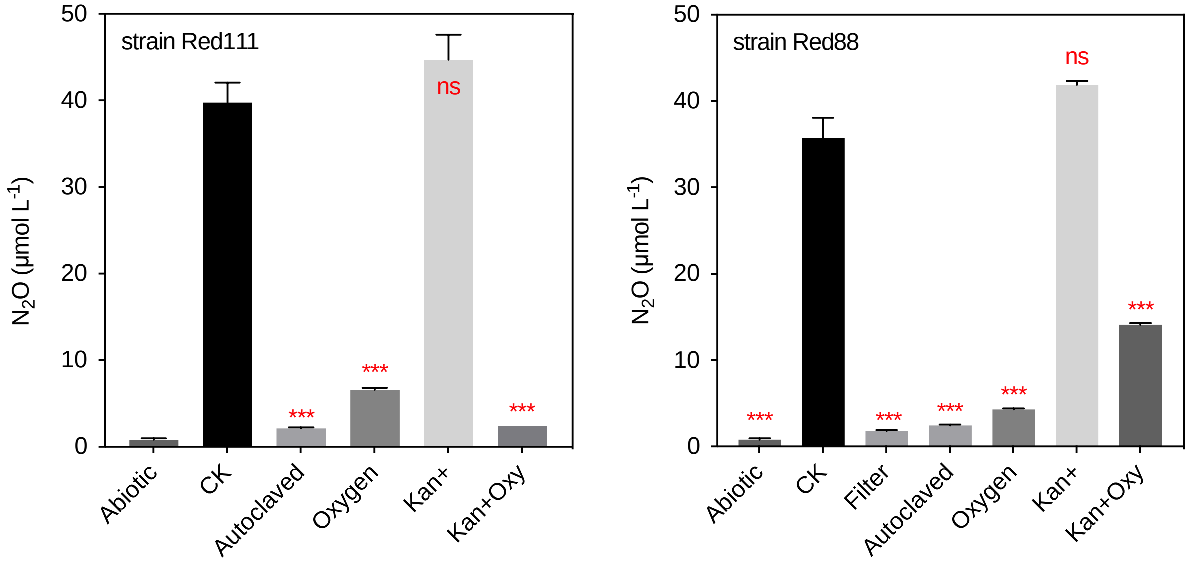

Fig. S5 N_2O production from the added SNP in the medium driving by strains *G. terrae* Red111 (**a**) and *O. rubra* Red88 (**b**) under different treatments: Abiotic, bacterial-free medium; CK, bacteria containing medium; Filter, filtered bacteria containing medium using 0.2 μm syringe filters; Autoclaved, autoclaved bacteria-containing medium at 121 °C for 20 mins; Oxygen, bacteria-containing medium gassed air; Kan+, bacteria containing medium supplemented with 100 μg/mL kanamycin; Kan+Oxy, bacteria containing medium gassed air and supplemented with 100 μg/mL kanamycin. The default medium is NNFM_N with additional 80 μM SNP. Asterisks (***) indicate significant difference, $p < 0.001$, while ns indicates insignificant difference, $p > 0.05$. The values are shown as mean ± standard deviation (SD).

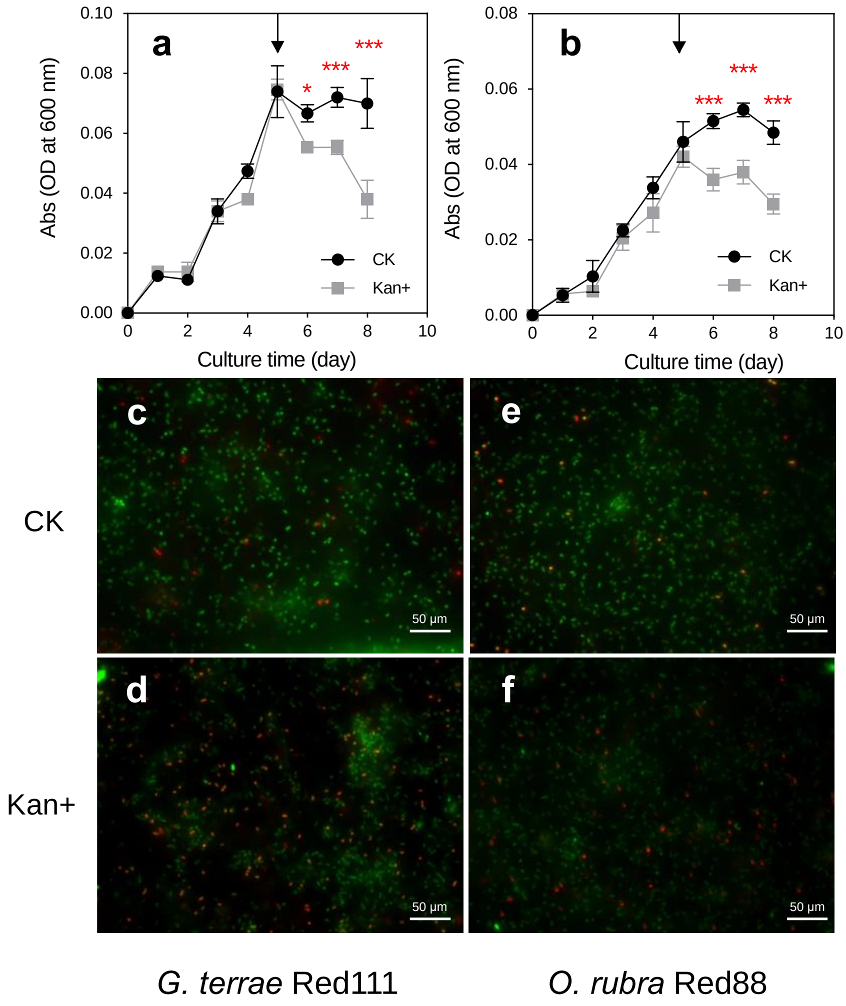

Fig. S6 The growth curves (**a**,**b**) and cell viability (**c**-**f**) of *O. rubra* Red88 and *G. terrae* Red111 with or without kanamycin (100 ug/mL) addition. CK indicates control experiments. Arrows indicate the time when kanamycin (100 ug/mL) was added to the medium. Asterisks indicate the significant difference, * p < 0.05, *** p < 0.001. The values are shown as mean ± standard deviation (SD). The cell viability was observed using a fluorescence microscopy after staining bacteria by a Live/Dead Cell Viability Assay Kit using the 7th-day samples. The green color labels the live cells, while the red color labels the dead cells.

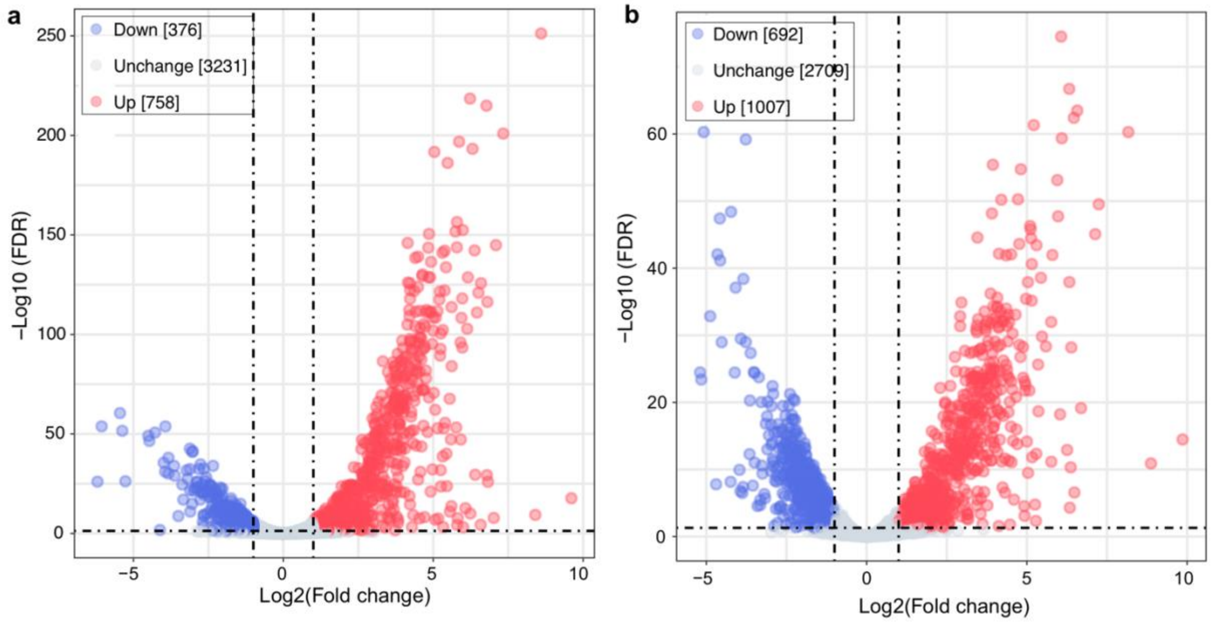

Fig. S7 Volcano plot showing the differentially expressed genes of the transcriptomic analysis in strain *Geomonas* sp. Red32 under 2 mM NO_2^- (**a**) and 8 mM NO_3^- (**b**) inductions. The abscissa shows the value of log2 of fold change, while the ordinate shows the value of −log10 of false discovery rate (adjusted *p* value). The vertical dotted lines represent 2-fold change threshold, while the horizontal line represents the adjusted *p* value of 0.05. Every gene is represented by a point, red points (right panel) represent significantly up-regulated genes (fold change > 2 and adjusted *p* value < 0.05), while blue points (left panel) represent significantly down-regulated genes (fold change < 0.5 and adjusted *p* value < 0.05), gray points (at the middle) represent nonsignificant or low differentially expressed genes (0.5 < fold change < 2 or adjusted *p* value > 0.05). Numbers in the square brackets represent the total number of genes belonging to up/down-regulated and unchanged groups.

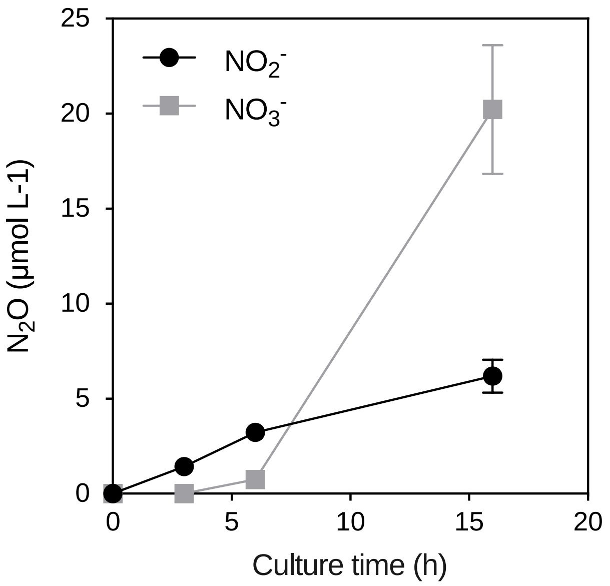

Fig. S8 N_2O production from NO_2^- (black circles) and NO_3^- (grey squares) reduction processes driving by strain *Geomonas* sp. Red32 at different culture time. The start time is from the NO_2^- (2 mM) and NO_3^- (8 mM) additions to the medium. All the culture conditions are completely identical to the transcriptomic study as described in the main text. The values are shown as mean ± standard deviation (SD).

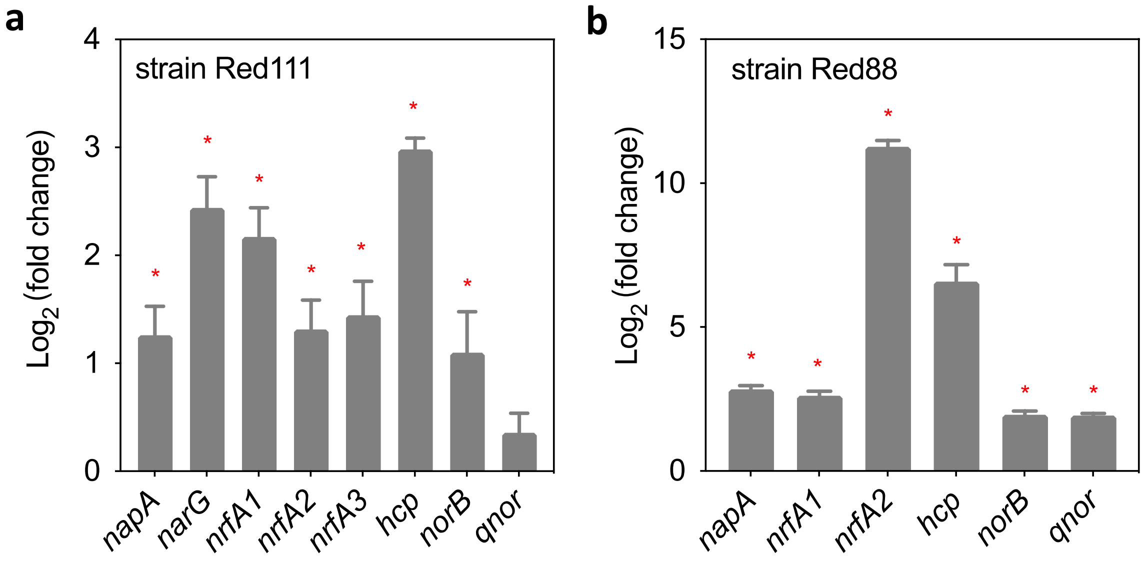

Fig. S9 The expression levels of representative genes related to DNRA and nitrosative stress processes from RT-qPCR in strains *G. terrae* Red111 (**a**) and *O. rubra* Red88 (**b**) with the SNP induction for 4h. Asterisk indicates significant difference, $p < 0.05$. The values are shown as mean ± standard deviation (SD). The locus tags of these representative genes are the same as described in Fig. S16b.

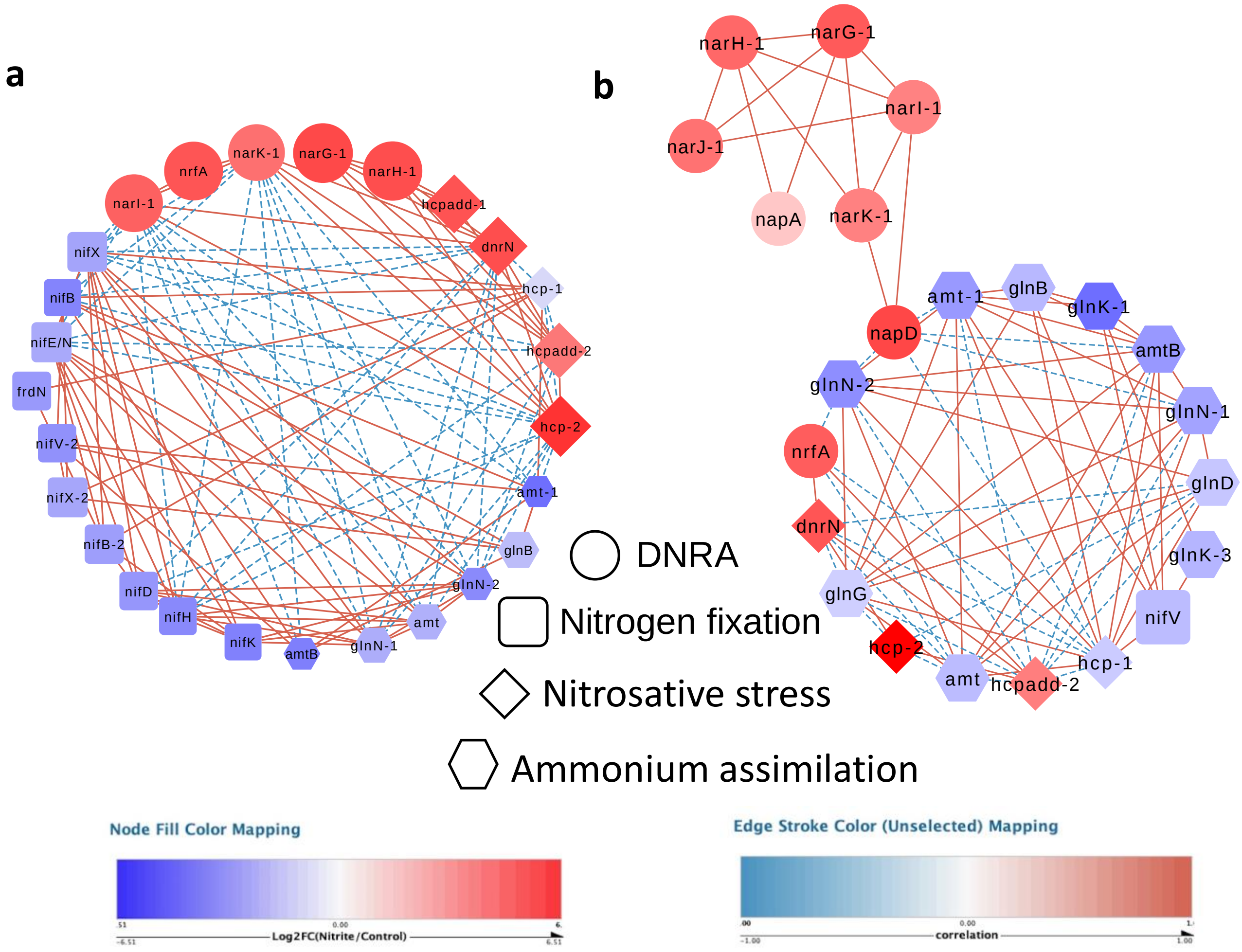

Fig. S10 Co-expressional network analysis of all selected genes related to nitrogen metabolism based on the transcriptomic results. **a**. NO_2^- treated condition; **b**. NO_3^- treated condition. Nodes represent the genes selected from nitrogen metabolism pathways with significant expression difference. Edges indicate the interaction between different genes with |Pearson correlation coefficient| > 0.9. Nodes with different shapes and sizes indicate the pathways and expression level, respectively, of target genes. Red nodes indicate up-regulated genes, while blue nodes indicate down-regulated genes. Red edges indicate positive correlations, while blue edges indicate negative correlation.

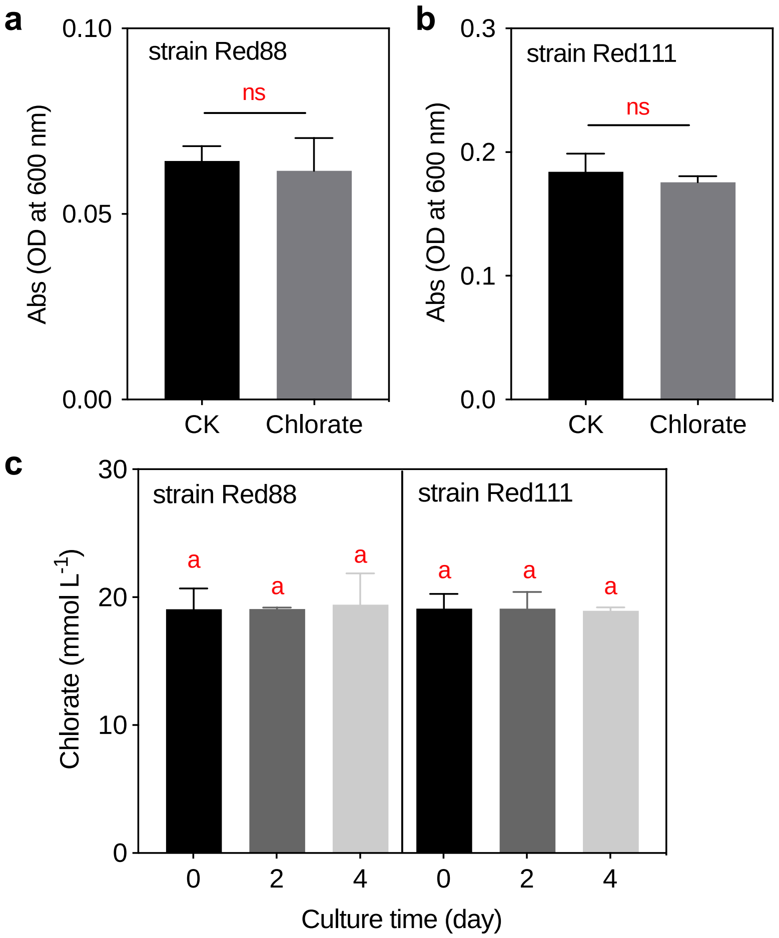

Fig. S11 The effect of chlorate on strains *G. terrae* Red111 and *O. rubra* Red88. **a-b**, the biomass of strains Red88 and Red111 with and without chlorate (20 mM) addition. **c**, the concentration of chlorate in the bacteria-containing medium at different culture time. The unchanged concentrations of chlorate indicate the inability of strains Red111 and Red88 in chlorate utilization. ns and the same letters ("a" in red) on the bars indicate insignificant differences (p > 0.05) of all comparisons. The values are shown as mean ± standard deviation (SD).

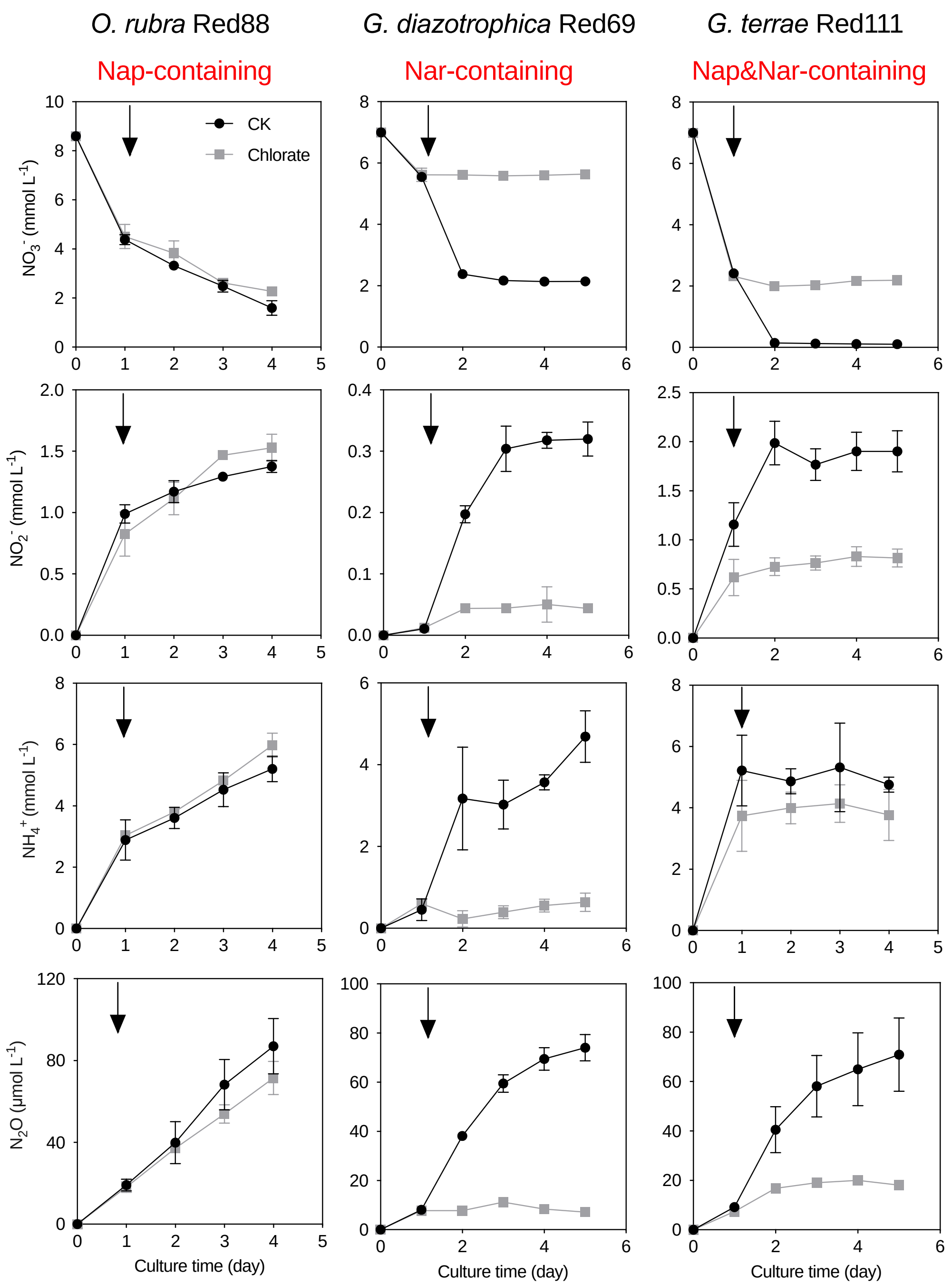

Fig. S12 The effect of chlorate on DNRA activities of strains *O. rubra* Red88 (left panel), *G. diazotrophica* Red69 (middle panel) and *G. terrae* Red111 (right panel). The DNRA activity is represented by the dynamic of NO_3^-, NO_2^-, NH_4^+, and N_2O concentrations in the cultures after NO_3^- (8 mM) addition. CK represents the negative control without chlorate addition (black circles), chlorate represents the treated group with chlorate (20 mM) addition (grey squares). Arrows indicate the time that chlorate was added to the medium. The values are shown as mean ± standard deviation (SD).

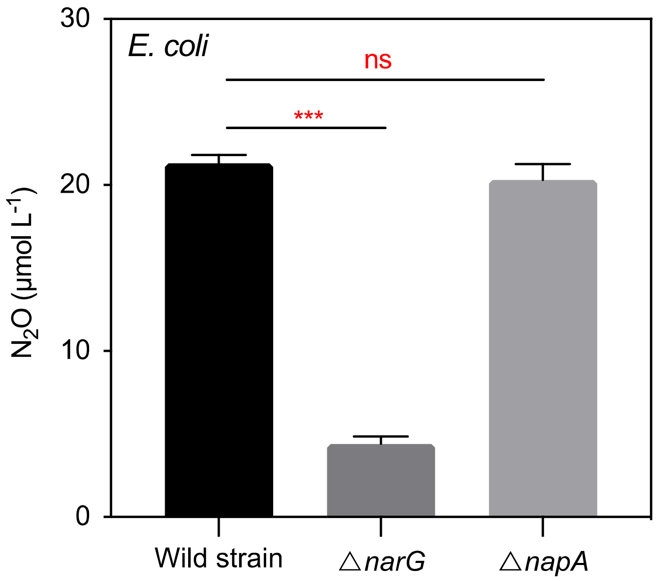

Fig. S13 N_2O production from the NO_2^- reduction process driving by strain *Escherichia coli* K-12 MG1655 (wild) and its *narG* (Δ*narG*) and *napA* (Δ*napA*) deletion mutants. All strains were anaerobically cultured using LB medium supplemented with 2 mM NO_2^- at 37 °C. Asterisks (***) indicate significant difference, $p < 0.001$, while ns indicates insignificant difference, $p > 0.05$. The values are shown as mean ± standard deviation (SD).

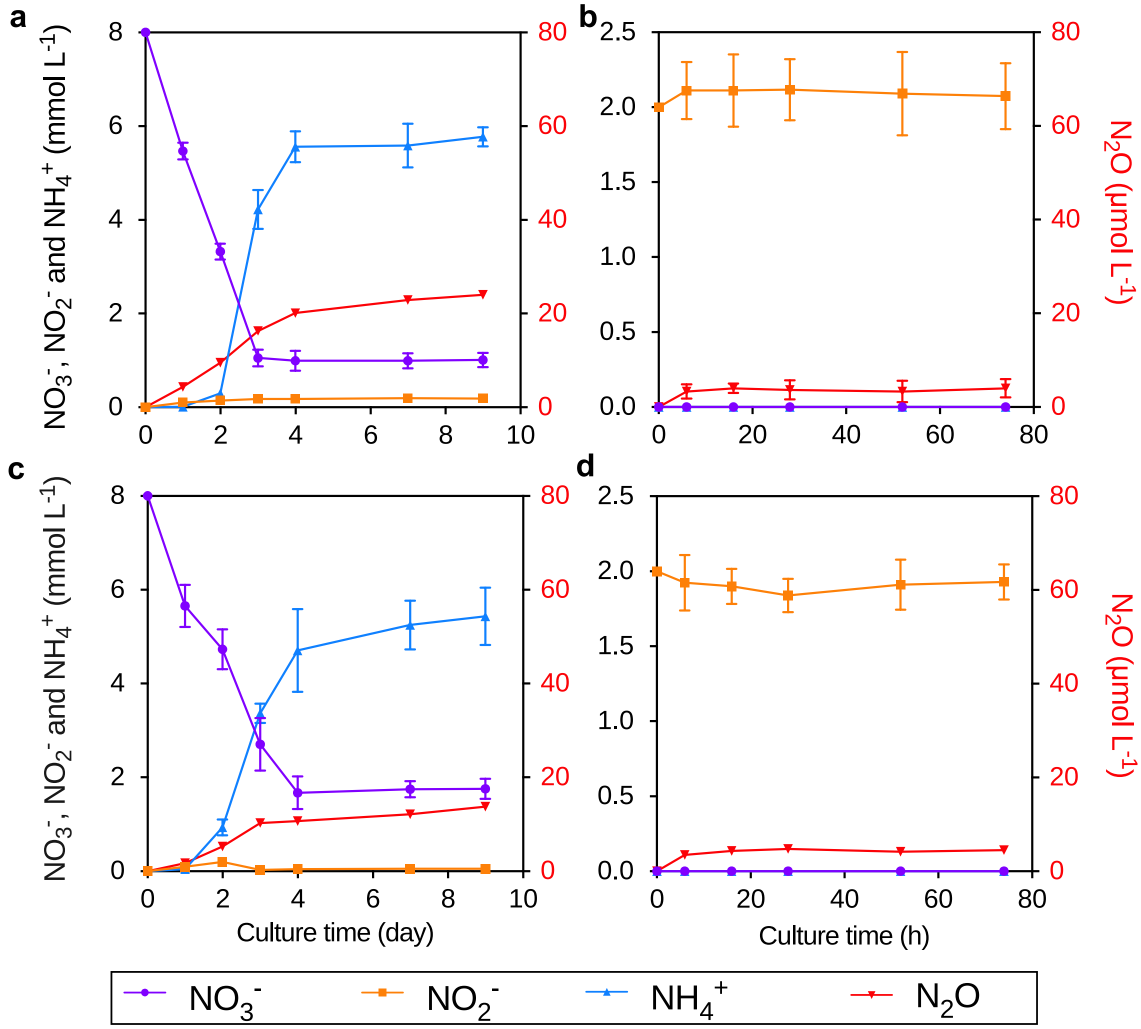

Fig. S14 Dissimilatory NO_3^- and NO_2^- reduction by two *Oryzomonas* strains Red96 (**a,b**) and Red100 (**c,d**). The start time was from the bacterial inoculation for the NO_3^- reduction process, while from the NO_2^- addition to the medium for the NO_2^- reduction process. The values are shown as mean ± standard deviation (SD).

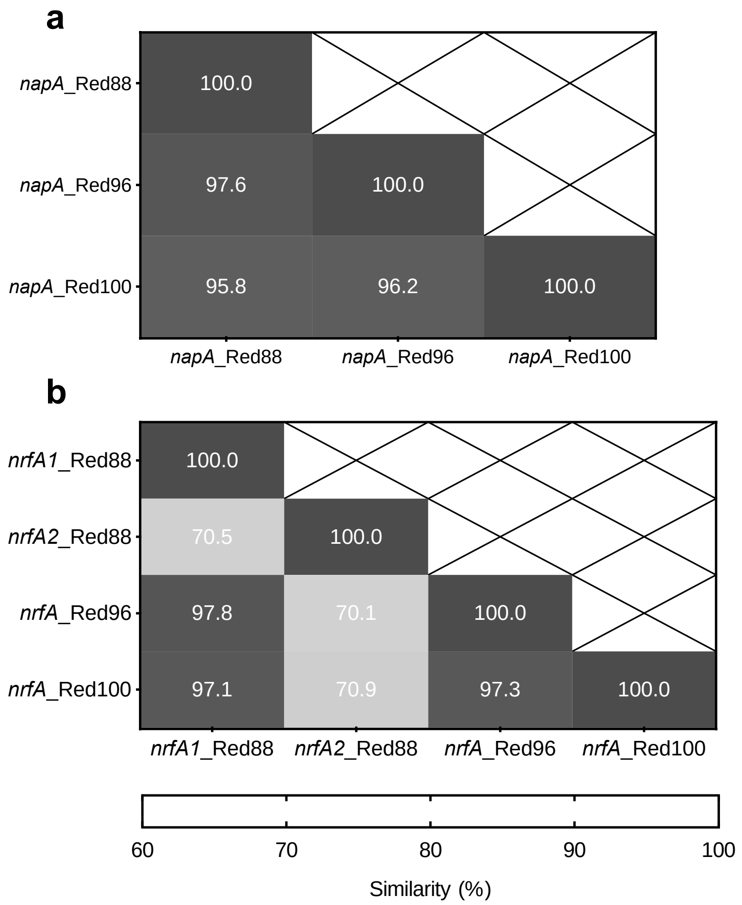

Fig. S15 The sequence similarity of DNRA-related genes in three *Oryzomonas* strains, *O. rubra* Red88, *O. japonica* Red96 and *O. sagensis* Red100. **a**, the *napA* gene similarity. **b**, the *nrfA* gene similarity. The similarity values of every pair of gene comparisons are denoted by the grey bar. The locus tags of these genes are the same as described in Fig. S17b.

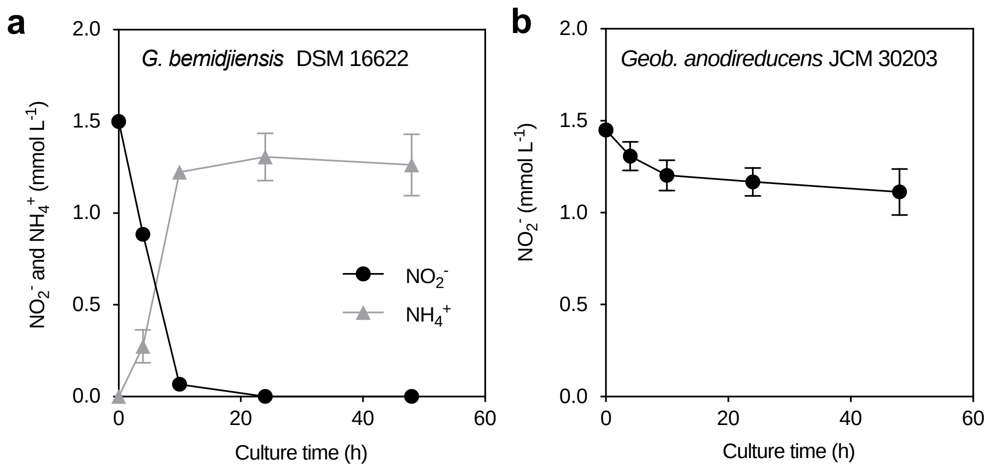

Fig. S16 Dynamics of NO_2^- (black circles) and NH_4^+ (grey triangles) concentrations in the cultures inoculated with *Geomonas bemidjiensis* DSM 16622 (**a**) and *Geobacter anodireducens* JCM 30203 (**b**). The start time is from the NO_2^- addition to the medium. No NH_4^+ was detected in the cultures with strain JCM 30203. The values are shown as mean ± standard deviation (SD).

a

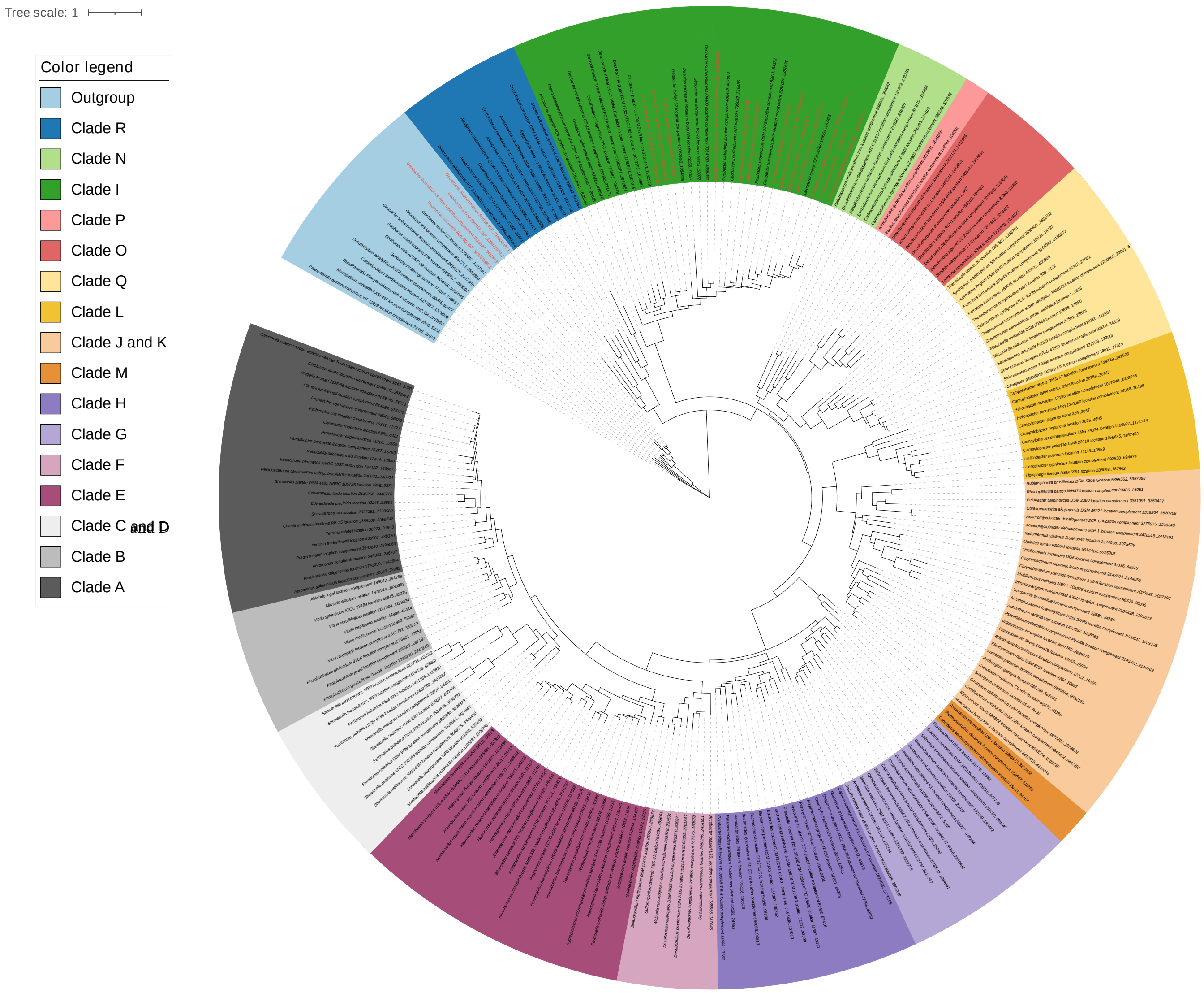

Tree scale: 1
Color legend
Outgroup
Clade R
Clade N
Clade I
Clade P
Clade O
Clade Q
Clade L
Clade J and K
Clade M
Clade H
Clade G
Clade F
Clade E
Clade C and D
Clade B
Clade A

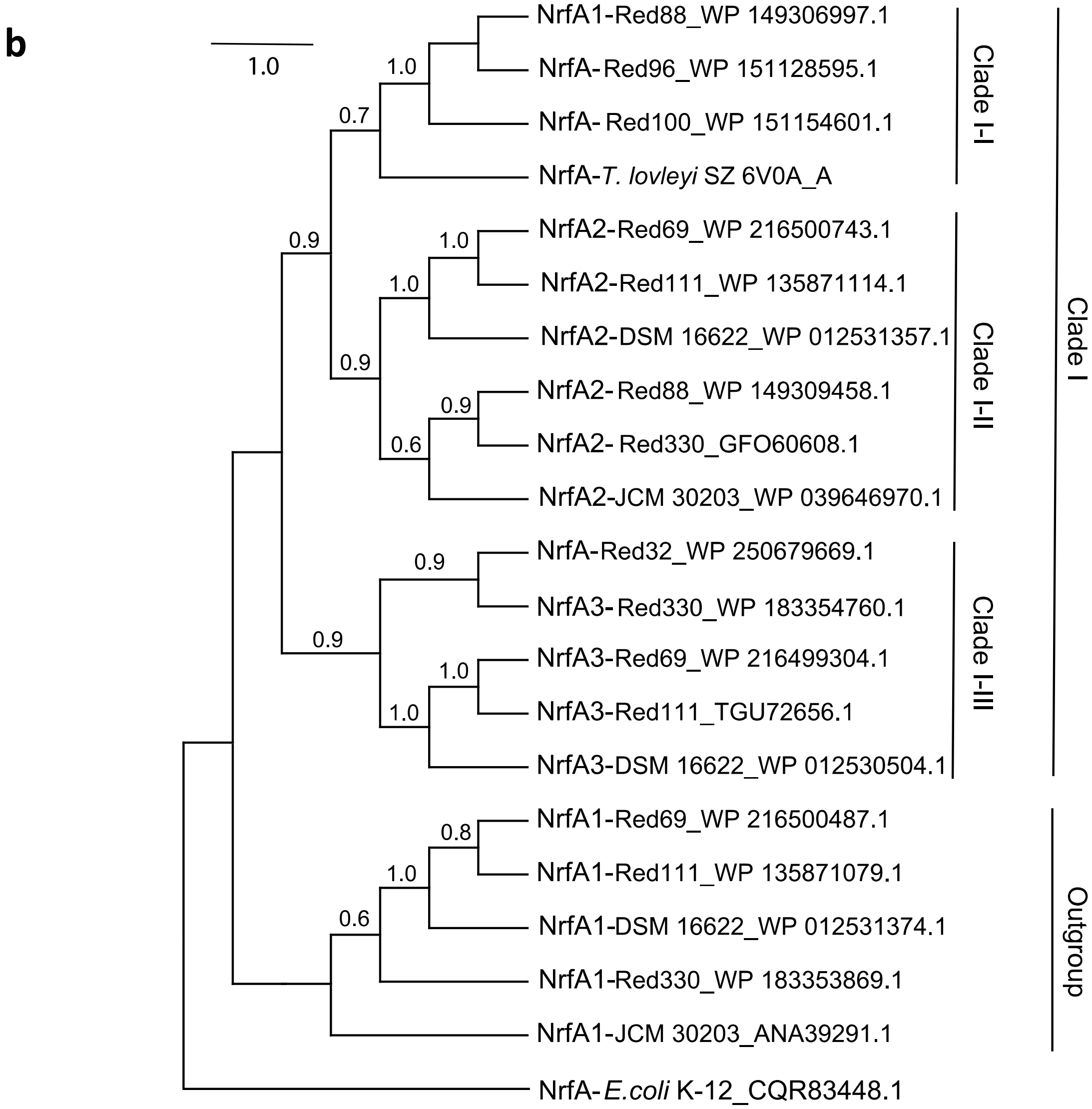

Fig. S17 Maximum-likelihood phylogenetic trees based on the NrfA amino acid sequences. **a**, the global tree based on 247 representative NrfA amino acid sequences showing the phylogenetic diversity of NrfA proteins. The 19 NrfA clades are labeled A to R and outgroup using different colors, as reported in ref(11, 12). Red fonts, in the clade I and outgroup, indicate the NrfA in studied *Geobacterales* strains. **b**, the simplified tree based on 20 NrfA amino acid sequences showing the phylogenetic positions of NrfA in studied *Geobacterales* strains. The subsets (I, II, and III) of clade I were proposed to distinguish NrfA proteins in the same bacterial strains. The NrfA in strain *E. coli* K-12 was used as the outgroup. Both phylogenetic trees were based on 1000 replications. The scale bar represents 1.0 substitution per amino acid position. Accession numbers or the locations in the genomes of NrfA amino acid sequences used in this study are given after the strain names in both trees. The IQ-TREE parameter for tree constructions is file_name -m MFP -b 1000 -redo.

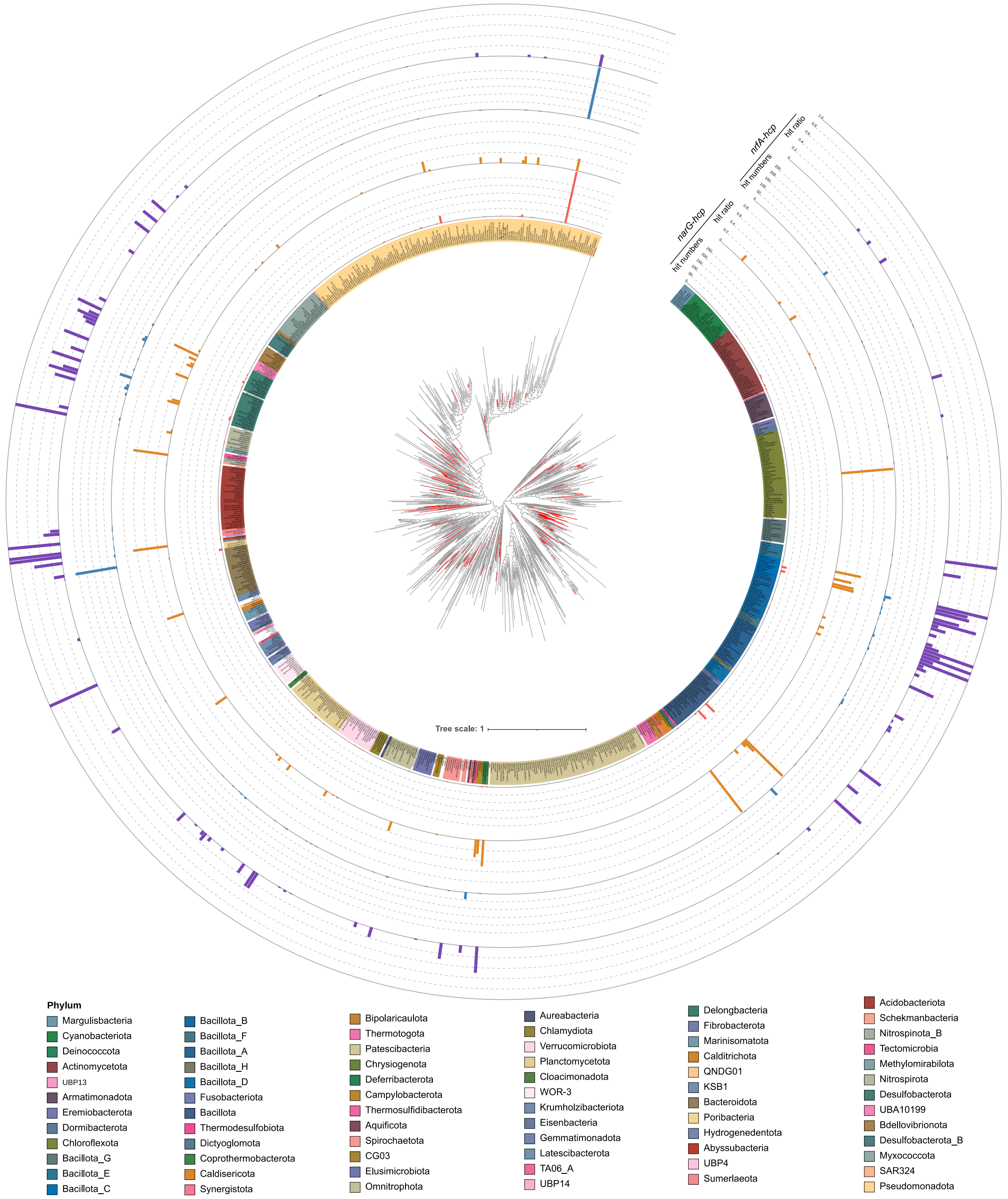

Fig. S18 Genome-based phylogenetic tree of all bacterial phyla presents in the Genome Taxonomy Database (GTDB) (Release RS95). Red branches indicated the bacterial groups at order levels contain the gene combinations *narG-hcp* or *nrfA-hcp*. The inner two rings show the hit numbers and hit ratios of *narG-hcp* gene combination, while the out two rings represent the hit numbers and hit ratios of *nrfA-hcp* gene combination. The phylogenetic tree was constructed by AnnoTree and visualized using iTOL. The raw data used in these rings are presented in Table S5.

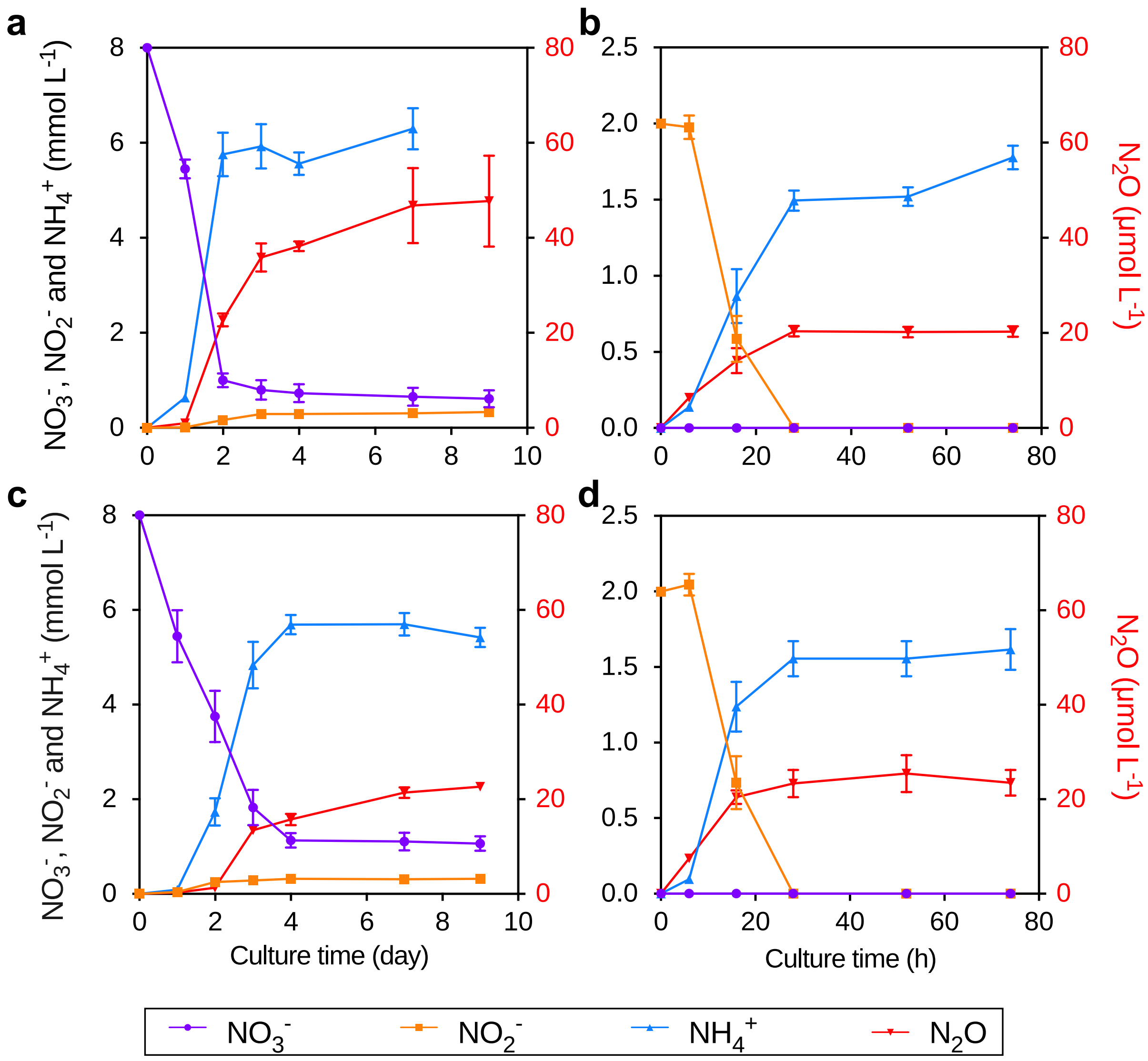

Fig. S19 Dissimilatory NO_3^- and NO_2^- reduction by two *Geomonas* strains Red69 (**a,b**) and Red330 (**c,d**). The start time of these Fig. was from the bacterial inoculation for the NO_3^- reduction process, while from the NO_2^- addition to the medium for the NO_2^- reduction process. The values are shown as mean ± standard deviation (SD).

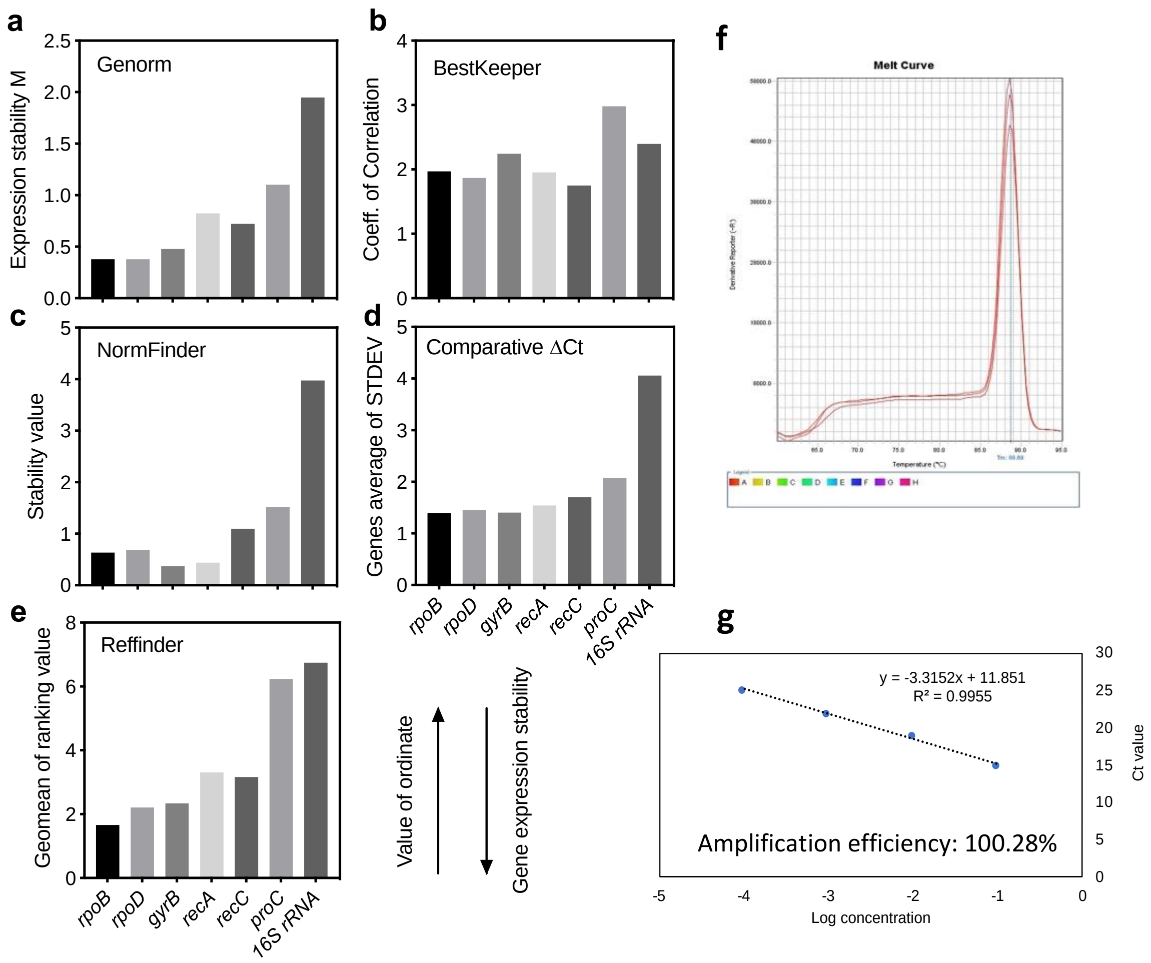

Fig. S20 Reference genes selection for RT-qPCR. **a-e**, the stability rank of seven candidate reference genes using different tools. The minimum value of the ordinate indicates the gene with the most stable expression. **f-g**, the melt curve and amplification efficiency from RT-qPCR of the selected reference gene *rpoB* with the primer pair used in this study.

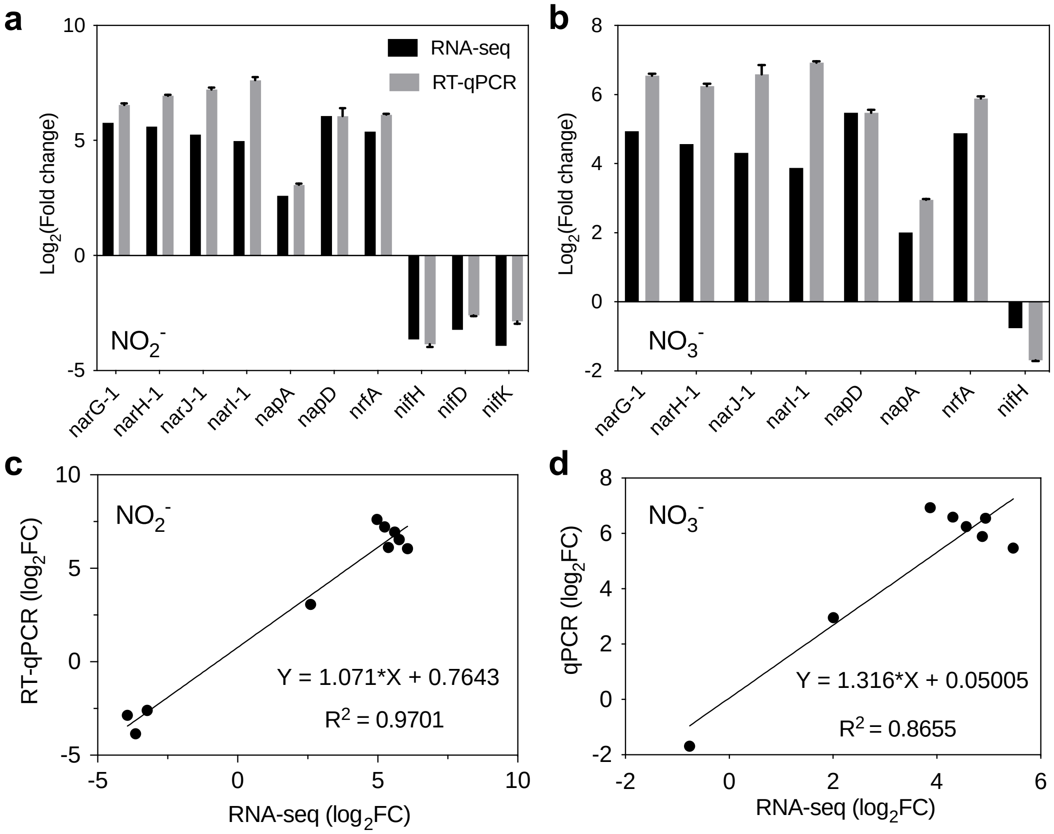

Fig. S21 Gene expression quantify by transcriptomic analysis and RT-qPCR and their correlation with 10 functional genes related to the nitrogen metabolism process in strain *Geomonas* sp. Red32 under NO_2^- and NO_3^- treatments. **a-b**, the expression levels of selected genes from transcriptomic analysis (RNA-seq, black bars) and RT-qPCR (grey bars) in strain *Geomonas* sp. Red32 with NO_3^- (8 mM, a) and NO_2^- (2 mM, b) inductions. The locus tags of these genes are the same as described in Fig. S17b. **c-d**, the correlation between transcriptomic data (RNA-seq) and RT-qPCR data. The lines indicate the best-fit line based on the simple linear regression model.
